# *Helicobacter pylori* infection induces STAT3 phosphorylation on Ser727 and autophagy in human gastric epithelial cells and mouse stomach

**DOI:** 10.1038/s41598-020-72594-3

**Published:** 2020-09-24

**Authors:** Juan-Yu Piao, Su-Jung Kim, Do-Hee Kim, Ji Hyun Park, Sin-Aye Park, Hyeong-jun Han, Hye-Kyung Na, Kichul Yoon, Ha-Na Lee, Nayoung Kim, Ki Baik Hahm, Young-Joon Surh

**Affiliations:** 1 Tumor Microenvironment Global Core Research Center, College of Pharmacy, Seoul National University, 1 Gwanak-ro, Gwanak-gu, Seoul, 08826 South Korea; 2 Department of Chemistry, College of Convergence and Integrated Science, Kyonggi University, Suwon, Gyeonggi-do 16227 South Korea; 3 Department of Internal Medicine and Liver Research Institute, Seoul National University College of Medicine, Seoul, 03080 South Korea; 4 Department of Biomedical Laboratory Science, College of Medical Sciences, Soonchunhyang University, Asan, 31538 South Korea; 5 Department of Food Science and Biotechnology, College of Knowledge-Based Services Engineering, Sungshin Women’s University, Seoul, 01133 South Korea; 6 Department of Internal Medicine and Digestive Disease Research Institute, Wonkwang University Sanbon Hospital, Gunpo, Gyeonggi-do 15865 South Korea; 7 Laboratory of Immunology, Center for Drug Evaluation and Research, Food and Drug Administration, Silver Spring, MD 20993 USA; 8 Digestive Disease Center, CHA University Bundang Medical Center, Seongnam, Gyunggi-do 13496 South Korea; 9 Department of Molecular Medicine and Biopharmaceutical Sciences, Graduate School of Convergence Science and Technology, Seoul National University, Seoul, 08826 South Korea

**Keywords:** Cancer, Molecular medicine

## Abstract

*Helicobacter pylori* (*H. pylori*) infection is considered as one of the principal risk factors of gastric cancer. Constitutive activation of the signal transducer and activator of transcription 3 (STAT3) plays an important role in inflammation-associated gastric carcinogenesis. In the canonical STAT3 pathway, phosphorylation of STAT3 on Tyr705 is a major event of STAT3 activation. However, recent studies have demonstrated that STAT3 phosphorylated on Ser727 has an independent function in mitochondria. In the present study, we found that human gastric epithelial AGS cells infected with *H. pylori* resulted in localization of STAT3 phosphorylated on Ser727 (P-STAT3^Ser727^), predominantly in the mitochondria. Notably, *H. pylori*-infected AGS cells exhibited the loss of mitochondrial integrity and increased expression of the microtubule-associated protein light chain 3 (LC3), the autophagosomal membrane-associated protein. Treatment of AGS cells with a mitophagy inducer, carbonyl cyanide 3-chlorophenylhydrazone (CCCP), resulted in accumulation of P-STAT3^Ser727^ in mitochondria. In addition, the elevated expression and mitochondrial localization of LC3 induced by *H. pylori* infection were attenuated in AGS cells harboring STAT3 mutation defective in Ser727 phosphorylation (S727A). We also observed that both P-STAT3^Ser727^ expression and LC3 accumulation were increased in the mitochondria of *H. pylori*-inoculated mouse stomach.

## Introduction

It is well known that the signal transducer and activator of transcription 3 (STAT3) functions as a nuclear transcription factor to regulate expression of target genes involved in cell proliferation, survival and transformation^1^. It has been reported that STAT3 is constitutively activated in human gastric adenomas, which is associated with poor prognosis^2^. *Helicobacter pylori* (*H. pylori*), a gram-negative bacterial pathogen, has been implicated in the development of gastric cancer. It has been well documented that *H. pylori* infection causes activation of STAT3 which is implicated in gastric cancer initiation and progression^3–6^. Our previous study demonstrated that *H. pylori* infection induced phosphorylation and nuclear translocation of STAT3 in cultured human gastric AGS cells and mouse stomach in vivo^7^.

One of the essential events in activation of STAT3 is phosphorylation at its Tyr705 residue. The tyrosine phosphorylated STAT3 (P-STAT3^Y705^) forms a dimer which translocates to nucleus and functions as a transcription factor. However, STAT3 undergoes phosphorylation on Ser727 as well, which is speculated to be involved in maximal activation of this transcription factor^8^. Notably, STAT3 was found to be constitutively phosphorylated on both Tyr705 and Ser727 in acute myeloid leukemia^9^. Sakaguchi et al. observed aberrant phosphorylation of STAT3 on Ser727 in melanoma cells irrespective of Tyr705 phosphorylation and demonstrated a role for P-STAT3^Ser727^ in regulating survival of these cells^10^. Moreover, the Ser727 phosphorylation of STAT3 is associated with negative estrogen receptor status in infiltrating breast ductal carcinoma^11^, and also progression of cervical intraepithelial neoplasia^12^.

Unlike Tyr705, Ser727 phosphorylation results in localization of STAT3 predominantly in mitochondria where it regulates the electron transport chain activity^13,14^. In addition, mitochondrial STAT3 phosphorylated on Ser727 contributes to *ras*-dependent oncogenic transformation^15^. Yu et al*.* also demonstrated that the mitochondrial P-STAT3^Ser727^, not nuclear P-STAT3^Tyr705^, was involved in malignant transformation of the Barrett’s epithelial cells expressing oncogene *ras*^16^. Moreover, mitochondrial localization of P-STAT3^Ser727^ promotes breast cancer growth by controlling the electron transport chain activity and regulating the intracellular accumulation of reactive oxygen species (ROS)^17^. However, the role for P-STAT3^Ser727^ in gastric carcinogenesis and related pathogenesis has not been investigated comprehensively. This prompted us to explore the role of P-STAT3^Ser727^ in the host response to *H. pylori*-associated gastritis.

## Results

### *H. pylori* induces phosphorylation of STAT3 on Ser727 in cultured AGS cells, independently of Tyr705 phosphorylation

**Figure 1:**
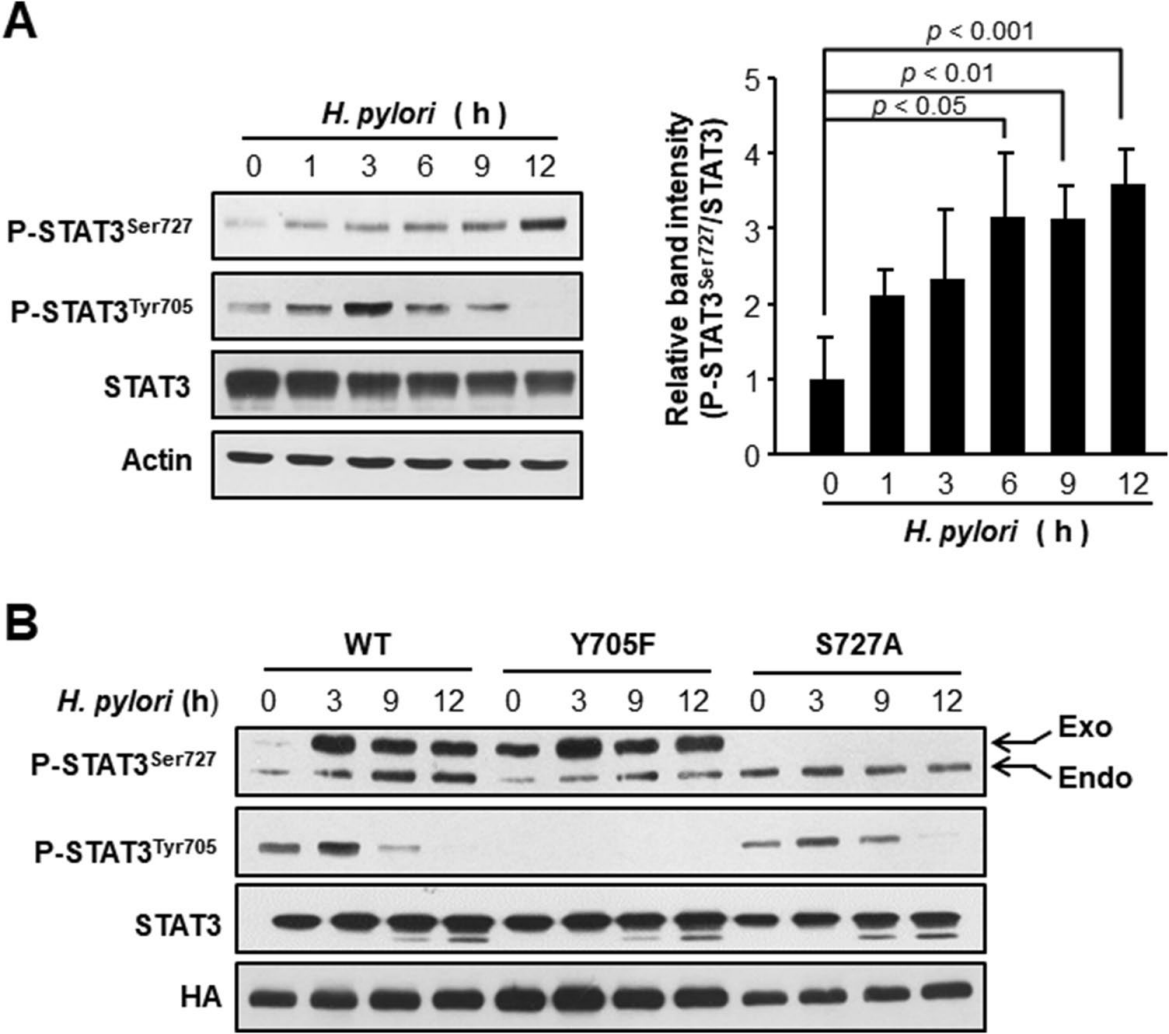
*H. pylori*-induced phosphorylation of STAT3 on Ser727 and Tyr705 in cultured AGS cells. (**A**) Western blot analysis of P-STAT3^Ser727^, P-STAT3^Tyr705^ and STAT3 in whole lysates. AGS cells were treated with 100 MOI *H. pylori* (ATCC43504) and harvested at indicated time points. The relative levels of P-STAT3^Ser727^ and STAT3 from three independent experiments are presented as means ± S.D. (**B**) Western blot analysis of P-STAT3^Ser727^, P-STAT3^Tyr705^ and STAT3 in whole lysates. AGS cells were transfected with WT and mutant vectors prior to *H. pylori* infection. The conditions for transient transfection and other experimental details are described in “Materials and methods”.

Constitutively elevated phosphorylation of STAT3 at the Tyr705 residue has been frequently observed in many different types of cancer cells and biopsies from cancer patients and is considered as an essential event for its transcriptional activity. However, recent studies have revealed that phosphorylation on Ser727 is also important for the oncogenic function of STAT3^11,12,15–17^. To determine whether *H. pylori* could induce STAT3 phosphorylation on Ser727, we conducted a time study upon *H. pylori* infection in cultured AGS cells. *H. pylori* infection induced STAT3 phosphorylation on Ser727 which exhibited a kinetic profile distinct from that for Tyr705 phosphorylation (Fig. 1A). To investigate whether or not phosphorylation of STAT3 at Tyr705 and Ser727 in *H. pylori*-infected AGS cells is causally linked, we performed site-directed mutagenesis targeting the tyrosine phosphorylation (Y705F) and serine phosphorylation (S727A) sites of the pCMV-STAT3-HA construct. AGS cells were then transfected with these phosphorylation-defective STAT3 mutant constructs prior to *H. pylori* infection. As illustrated in Fig. 1B, Tyr705 mutation did not influence phosphorylation of STAT3 on Ser727 and vice versa, suggesting that *H. pylori*-induced phosphorylation of STAT3 on Tyr705 and Ser727 occurs independently of each other.

### STAT3 phosphorylated on Ser727 predominantly localizes in mitochondria of *H. pylori*-infected AGS cells

**Figure 2:**
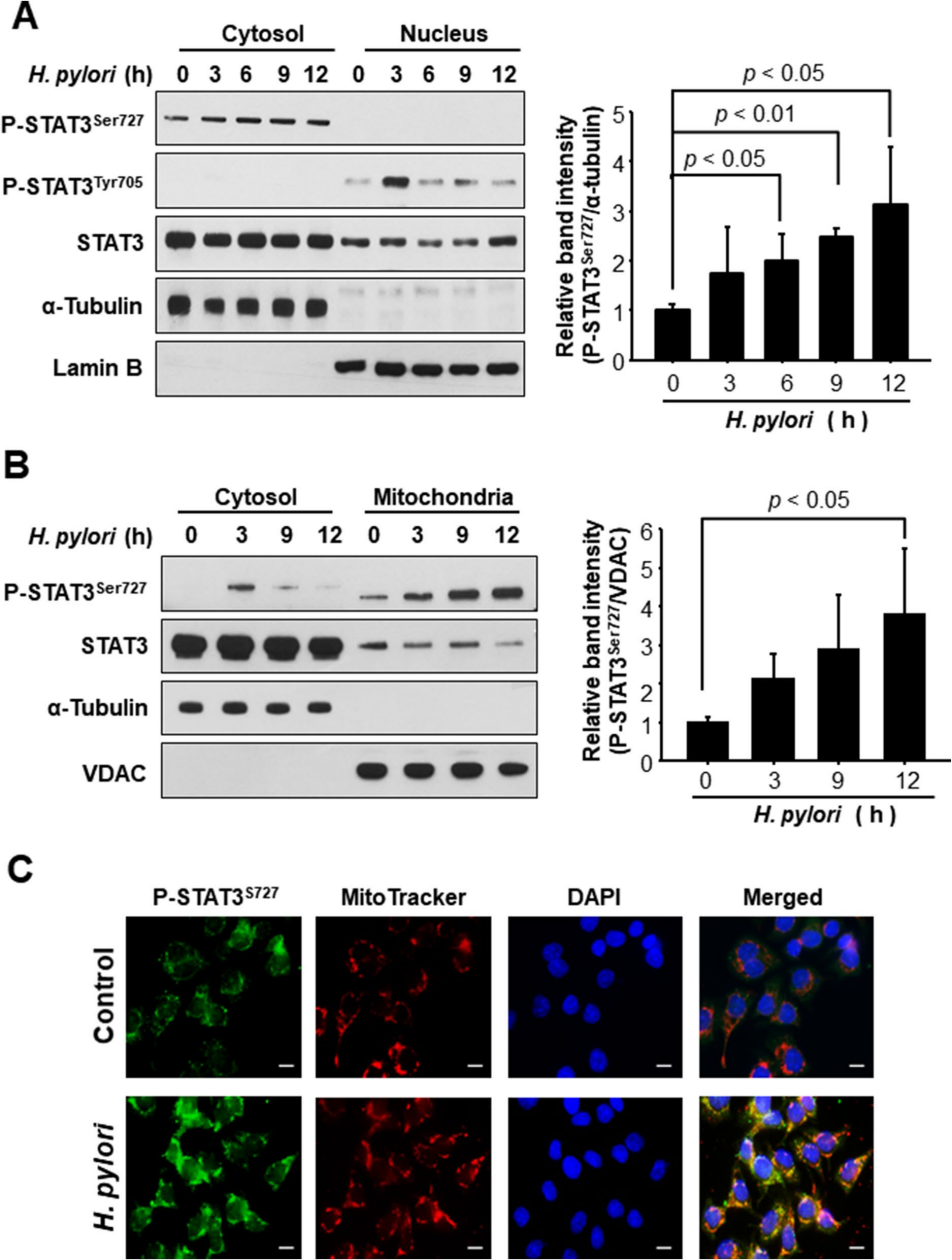
Comparison of subcellular localization of P-STAT3^Ser727^ in AGS cells infected with *H. pylori*. AGS cells were co-cultured with *H. pylori* and harvested at indicated time points. (**A**) Western blot analysis of P-STAT3^Ser727^, P-STAT3^Tyr705^ and STAT3 in cytosolic and nuclear extracts. Lamin B and α-tubulin were used as nuclear and cytoplasmic protein markers, respectively. (**B**) Western blot analysis of P-STAT3^Ser727^ and STAT3 in cytosolic and mitochondrial extracts. VDAC and α-tubulin are mitochondrial and cytoplasmic protein markers, respectively. (**C**) Verification of mitochondrial translocation of P-STAT3^Ser727^ upon *H. pylori* infection for 12 h. Mitotracker was used as a selective probe of mitochondria. Scale bar, 100 μm.

Next, we investigated the subcellular localization of P-STAT3^Ser727^ in *H. pylori*-infected AGS cells. According to Western blot analysis of the cytosolic and nuclear extracts, P-STAT3^Tyr705^ was detected in nucleus, whereas STAT3 phosphorylated on Ser727 was mainly present in cytosol (Fig. 2A), indicating that P-STAT3^Ser727^ is unlikely to function as a transcription factor. It has been reported that mitochondrial STAT3 promotes *ras*-dependent oncogenic transformation, which is largely dependent on Ser727 phosphorylation^15,16^. Therefore, we further fractionated the cytoplasm to get the mitochondrial fraction which was subjected to Western blot analysis. Notably, P-STAT3^Ser727^ predominantly localized in mitochondria of *H. pylori*-infected AGS cells (Fig. 2B), and this result was verified by the immunocytochemical analysis using a selective mitochondrial probe MitoTracker (Fig. 2C). Serine phosphorylated STAT3 has been reported to localize in nucleus^18,19^ as well as in cytosol and mitochondria. In our present study, Western blot analysis barely detected P-STAT3^S727^ in the nucleus (Fig. 2A), but immunofluorescence analysis (Fig. 2C) detected 4′,6-diamidino-2-phenylindole (DAPI)-stained P-STAT3^S727^, indicative of its nuclear localization although the extent was apparently much less evident than that of mitochondrial accumulation.

### *H. pylori* infection provokes mitochondrial damage and autophagy

**Figure 3:**
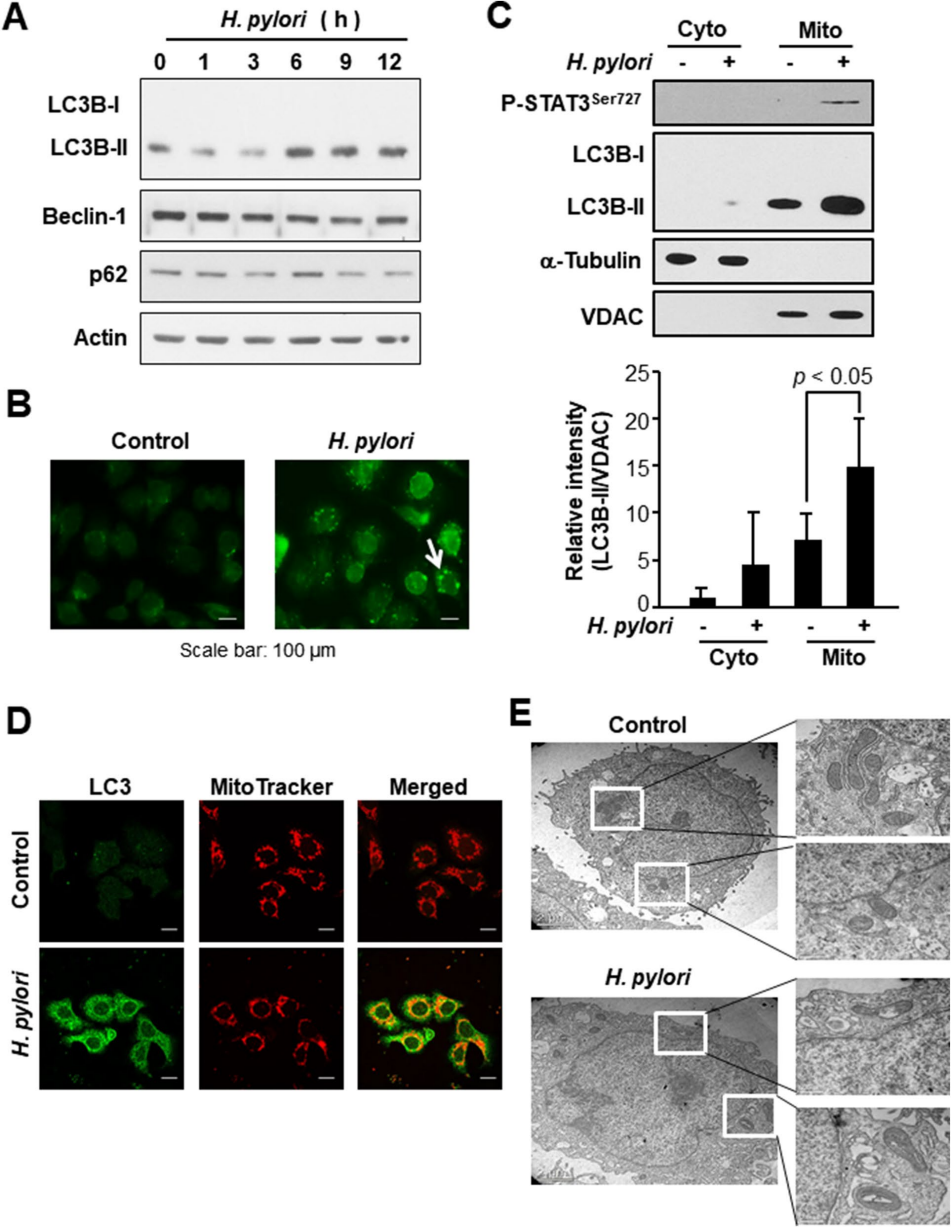
Mitochondrial accumulation of an autophagy marker in *H. pylori* infected AGS cells. (**A**) AGS cells were infected with *H. pylori* and harvested at indicated time points. Western blot analyses of LC3B, Beclin 1, p62 and actin in whole cell lysates. (**B**) Immunocytochemical analysis of LC3 in AGS cells infected by *H. pylori* for 12 h. The arrow indicates the LC3 dots. (**C**) Western blot analysis of P-STAT3^Ser727^ and LC3B in cytosolic and mitochondrial fractions of *H. pylori* infected AGS cells. VDAC and α-tubulin were used as mitochondrial and cytoplasmic protein markers, respectively. The relative expression levels of LC3B-II from three independent experiments are presented means ± S.D. (**D**) Immunocytochemical analysis of LC3 in AGS cells co-cultured with *H. pylori* for 12 h. MitoTracker is a selective probe of mitochondria. Scale bar, 100 μm. (**E**) Transmission electron microscopy image of control cells and *H. pylori*-infected cells.

Autophagy is an autodigestive process that degrades cellular organelles and proteins to maintain cellular homeostasis against environmental stress^20^. It has been reported that *H. pylori* induces autophagy via the virulence factor of VacA^21^. In agreement with this finding, expression of the autophagy marker, the microtubule-associated protein light chain 3 (LC3) B (Fig. 3A) and formation of LC3 dots (Fig. 3B) were increased by *H. pylori* infection in AGS cells. However, there was not much change in the expression of other autophagy markers, such as Beclin-1 and p62. Notably, the increased accumulation of autophagosomal membrane-associated protein LC3B was observed in the mitochondrial fraction of *H. pylori*-infected AGS cells, as assessed by Western blot (Fig. 3C) and immunocytochemical (Fig. 3D) analyses.

While the uninfected AGS cells maintain integrity of the mitochondria, *H. pylori*-infected AGS cells exhibited swelling of mitochondria as visualized by transmission electron microscopy (Fig. 3E). These results suggest that *H. pylori* infection causes mitochondrial damage, and the injured mitochondria are likely to be sequestered by autophagosomes which eventually fuse with the lysosomes for clearance.

### *H. pylori*-induced accumulation of LC3 is associated with STAT3 phosphorylation on Ser727

**Figure 4:**
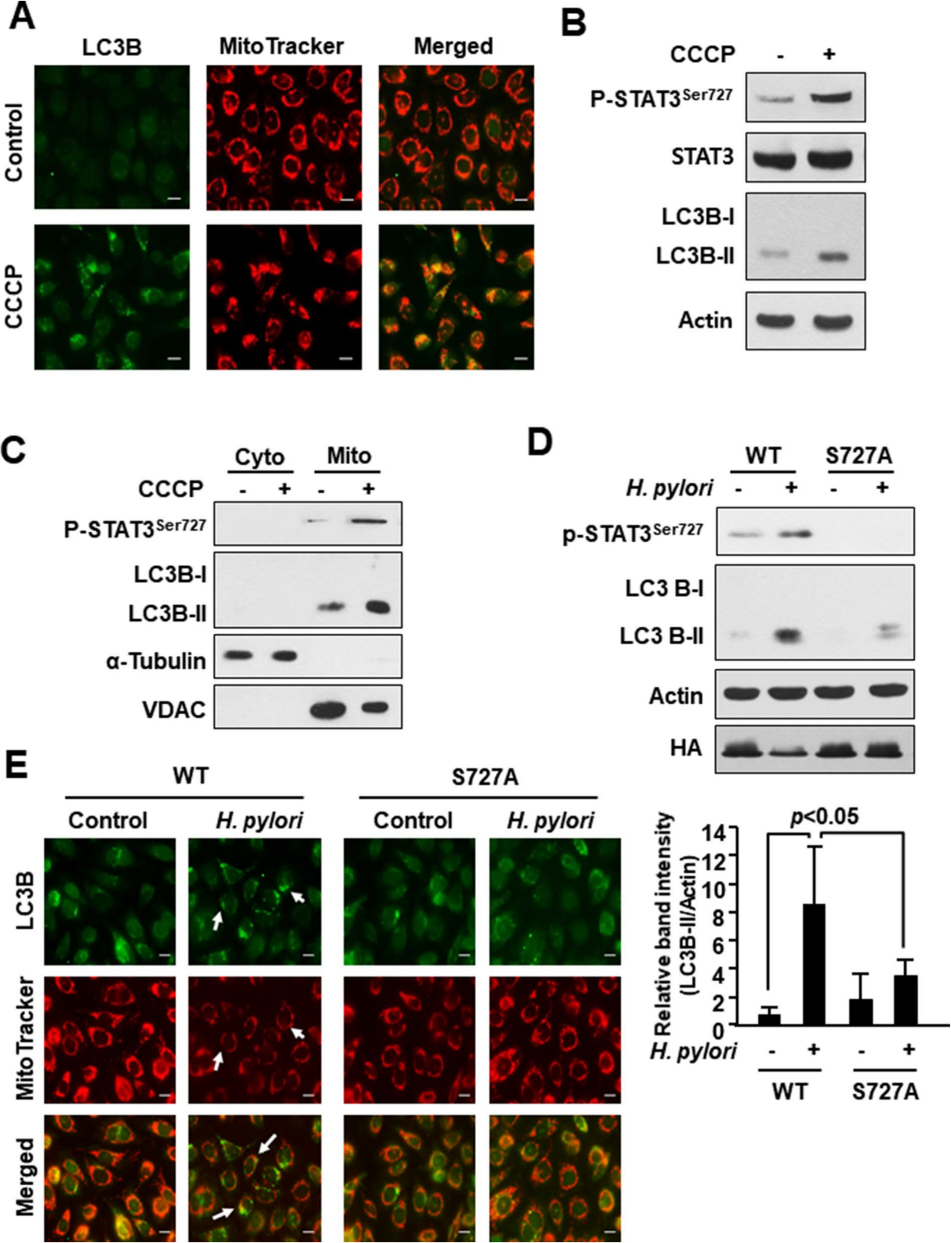
Role of P-STAT3^S727^ in accumulation of LC3 in mitochondria of *H. pylori* infected AGS cells. (**A**) Immunocytochemical analysis of LC3 co-localized with mitochondria. AGS cells were treated with vehicle (DMSO) or CCCP (1 μM) for 1 h. MitoTracker was used as a selective probe of mitochondria. Scale bar, 100 μm. (**B**) Western blot analysis of P-STAT3^Ser727^, STAT3, and LC3B in AGS cells treated with DMSO or 1 μM CCCP for 1 h. (**C**) AGS cells were treated with DMSO or CCCP (1 μM) for 1 h. Western blot analysis of P-STAT3^Ser727^ and LC3B in cytosolic and mitochondrial extracts. VDAC and α-tubulin are mitochondrial and cytoplasmic protein markers, respectively. (**D**) Western blot analysis of P-STAT3^Ser727^ and LC3B in AGS cells transfected with WT or serine dominant negative mutant vector. The relative expression levels of LC3 from three independent experiments are presented means ± S.D. (**E**) Immunocytochemical analysis of LC3B in WT and Ser727 mutant AGS cells with or without *H. pylori* infection. MitoTracker is a selective probe of mitochondria. Scale bar, 100 μm.

To determine whether P-STAT3^Ser727^ could be involved in clearance of damaged mitochondria by autophagy, we treated the AGS cells with a well-known mitophagy inducer carbonyl cyanide 3-chlorophenylhydrazone (CCCP). CCCP treatment dramatically increased co-localization of mitochondria with LC3B (Fig. 4A). In addition, the expression of P-STAT3^Ser727^ in whole (Fig. 4B) and mitochondrial (Fig. 4C) lysates was increased by CCCP treatment. Notably, CCCP–induced LC3B-II expression was abrogated in AGS cells transfected with the serine 727 mutant vector (Supplementary Fig. 1). We then investigated the possible role of Ser727 phosphorylated STAT3 in *H. pylori-*induced LC3 accumulation in the mitochondria of AGS cells. Those cells transiently transfected with phosphorylation defective S727A vector exhibited a markedly reduced expression level of LC3B-II upon *H. pylori* infection (Fig. 4D). Furthermore, LC3 dot formation and decreased mitochondria mass observed in wild-type cells following *H. pylori* infection were not evident in the mutant cells (Fig. 4E). These results, taken all together, suggest that STAT3 phosphorylation on Ser727 may account for *H. pylori*-induced autophagic removal of damaged mitochondria.

**Figure 5:**
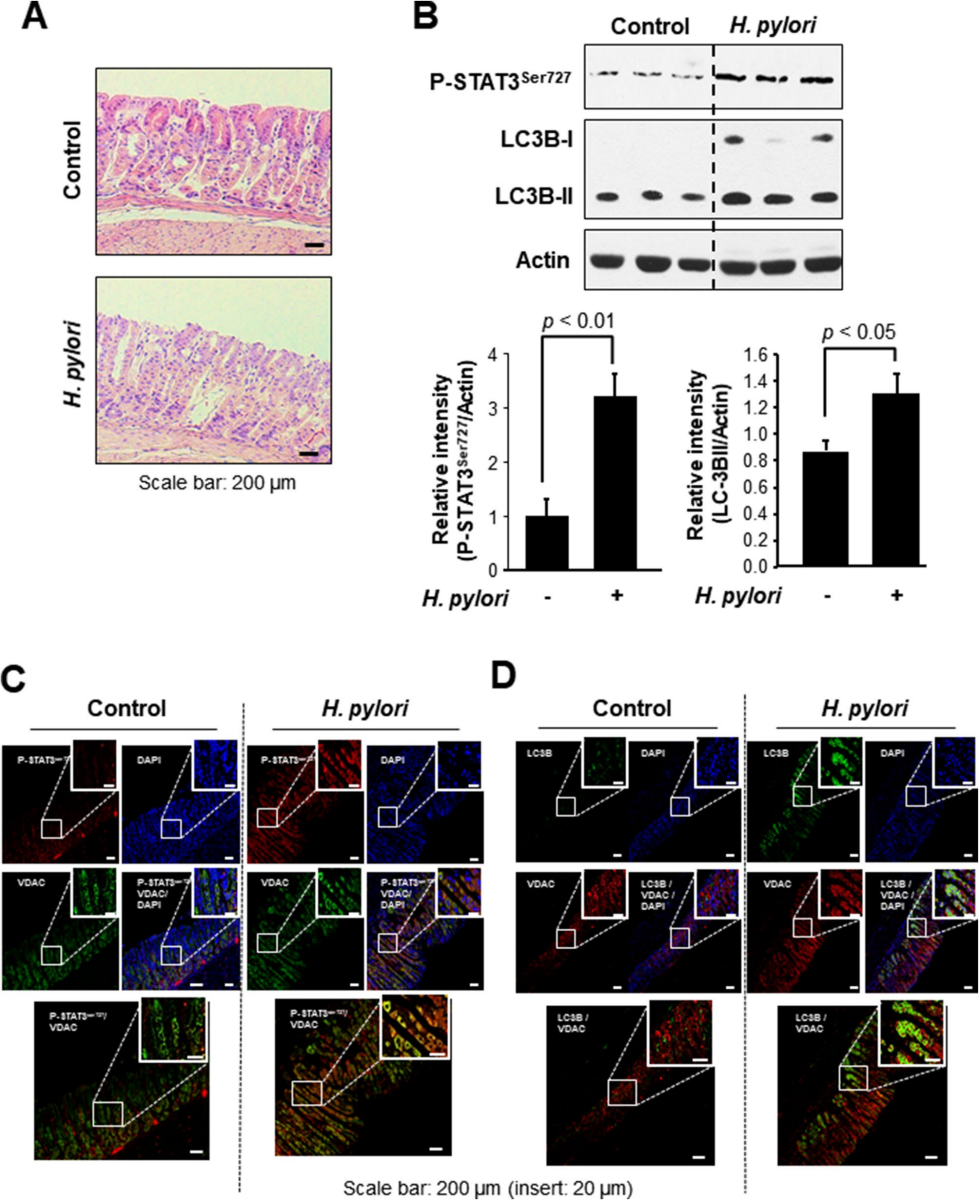
STAT3 phosphorylation on Ser727 and LC3B expression in mouse stomach infected with *H. pylori*. (**A**) H&E staining of *H. pylori*-infected mouse stomach tissue. (**B**) Western blot analysis of P-STAT3^Ser727^ and LC-3B in *H. pylori*-infected mouse stomach. The relative expression levels of P-STAT3^Ser727^ and LC-3B are presented means ± S.D. (**C**) Immunofluorescence analysis of P-STAT3^Ser727^ co-localized with a mitochondrial marker protein, VDAC. (**D**) Immunofluorescence analysis of LC3B co-localized with VDAC.

We also explored possible association between Ser727 phosphorylation of STAT3 and autophagy in a *H. pylori*-induced murine gastritis model. The *H. pylori*-induced gastritis was evidenced by elongation of gastric pits and loss of parental cells according to histological examinations (Fig. 5A). As shown in Fig. 5B, expression of both P-STAT3^Ser727^ and LC3B was enhanced in the gastric mucosa of *H. pylori* infected mice. Further, *H. pylori*-inoculated mouse stomach had elevated co-localization of P-STAT3^Ser727^ and the mitochondrial voltage-dependent anion channel (VDAC). Moreover, enhancement of LC3B localization was observed in the mitochondria of *H. pylori*-infected mouse mucosa (Fig. 5D).

## Discussion

STAT3 is a transcription factor activated by various external stimuli including cytokines and growth factors. Upon activation, STAT3 is phosphorylated on Tyr705 and translocates to nucleus where it regulates expression of target genes involved in cell proliferation, survival and so on^1,3,5–7,22^. Although phosphorylation of STAT3 on Tyr705 has been considered essential for its dimerization, nuclear translocation, transcriptional activity and oncogenic function, STAT3 phosphorylated on Ser727 is imported into mitochondria and regulates the mitochondrial electron transport chain activity and *ras*-dependent malignant transformation^15,16^. The phosphorylation at serine 727 also stimulated breast tumor growth by modulating the activity of complex I and the intracellular accumulation of ROS^17^.

The association between Tyr705 and Ser727 residues of STAT3 is controversial. Some studies demonstrated that STAT3 Ser727 phosphorylation negatively modulated Tyr705 phosphorylation^23,24^. In glioma-initiating cells, Tyr705 phosphorylation was independent of Ser727 phosphorylation while Ser727 phosphorylation was dependent on Tyr705 phosphorylation^25^. However, it has been reported that phosphorylation of STAT3 on Ser727 promotes prostate tumorigenesis, regardless of Tyr705 phosphorylation^26^. Our previous studies demonstrated that *H. pylori* infection gave rise to increased Tyr705 phosphorylation and nuclear translocation of STAT3 in cultured AGS cells and mouse stomach in vivo^7^. In the study reported here, we found that *H. pylori* infection induced phosphorylation of STAT3 on Ser727 as well, and the resulting P-STAT3^Ser727^ was detected predominantly in mitochondria and that nuclear translocation of P-STAT3^Tyr705^ and mitochondrial localization of P-STAT3^Ser727^ occurred, independently of each other in *H. pylori* infected human gastric cancer cells.

Autophagy is an autodigestive process, which removes unnecessary or dysfunctional components to maintain cellular homeostasis in response to nutrient stress^20^. During the process of autophagy, the LC3 is converted to the LC3-II form, recruiting autophagosomal membranes. Our present study reveals that *H. pylori*-induced accumulation of LC3-II in the mitochondria is likely to be mediated by P-STAT3^Ser727^. The involvement of STAT3 in regulating autophagy has received considerable attention in recent years^27–30^. Several studies have highlighted regulatory roles for ROS of mitochondrial origin in the induction of autophagy, especially the selective autophagic degradation of mitochondria, also known as mitophagy^17,31^. However, connections between mitochondrial STAT3 activities and ROS production are somewhat contradictory^32^.

It should be noted that no specific mitochondrial targeting signal sequence has been identified in STAT3 although the C-terminus has been shown to be required for its mitochondrial transport. STAT3 phosphorylated on Ser727 translocates to mitochondria which is known to be dependent on GRIM-19^33^, a component of complex I required for maintenance of the mitochondrial membrane potential^34,35^. The phosphorylation of STAT3 on Ser727 facilitates its interaction with GRIM-19 with a resultant translocation of STAT3 to the mitochondria where it induces an increase in ROS production and cell death^36^. In support of this speculation, we found a physical interaction between Ser727 phosphorylated STAT3 and GRIM-19 in mitochondria of *H. pylori*-infected cells (Supplementary Fig. 2A), and this binding was blunted when the Ser727 residue of STAT3 was replaced by alanine (Supplementary Fig. 2B). Furthermore, siRNA knockdown of GRIM-19 abolished *H. pylori*-induced localization of P-STAT3^Ser727^ in the mitochondria (Supplementary Fig. 2C) as well as expression of LC-3 (Supplementary Fig. 2D). This result suggests that *H. pylori*-induced mitochondrial translocation of P-STAT3^Ser727^ is mediated by GRIM-19.

Mitophagy, a specific form of autophagy, removes damaged mitochondria to prevent cytochrome *c* release into cytosol, thereby protecting against apoptotic cell death upon mild stress^37^. However, the (patho)physiologic significance of mitophagy is still obscure. Increased ROS production by impairment of mitochondrial electron transport can trigger inflammatory signaling. Accumulation of mitochondria-derived ROS then activates redox sensitive transcription factors, facilitating the production of anti-inflammatory or proinflammatory factors. Although mitophagy can specifically eliminate dysfunctional mitochondria to maintain cellular homeostasis, thereby preventing hyperinflammation caused by ROS, defective or overactivated mitophagy may aggravate the inflammation, provoking pathological conditions^38,39^.

It is well established that *H. pylori* infection induces inflammatory responses leading to an increased risk of gastric cancer. As shown in this study, *H. pylori*-infected mouse stomach mucosa exhibited elongation of gastric pit and loss of parental cells, reflecting inflammatory tissue damage. Furthermore, the levels of both P-STAT3^Ser727^ and LC3 were elevated in *H. pylori*-positive mouse stomach, suggesting the potential link between inflammation and autophagy. A prototypic pro-inflammatory enzyme, cyclooxygenase-2 (COX-2) is one of the downstream targets of the oncogenic transcription factors NF-κB and STAT3. Both *H. pylori* (Supplementary Fig. 3A) and the mitophagy inducer CCCP (Supplementary Fig. 3B) upregulated the expression of COX-2, indicative of association between *H. pylori*-promoted mitophagy and inflammatory response. Moreover, silencing of LC3 abrogated the *H. pylori*-induced COX-2 expression (Supplementary Fig. 3C). A similar result was achieved by use of an autophagy inhibitor (3-MA) (Supplementary Fig. 3D). Notably, *H. pylori*-induced enhancement of COX-2 expression was negated in AGS cells harbouring a serine to alanine mutation (S727A) of STAT3 (Supplementary Fig. 3E). *H. pylori*-induced COX-2 expression in AGS cells was attenuated by silencing of GRIM-19 (data not shown), suggesting that interaction of P-STAT3^Ser727^ with GRIM-19 may trigger inflammatory signaling in response to *H. pylori*-induced gastric inflammation.

**Figure 6:**
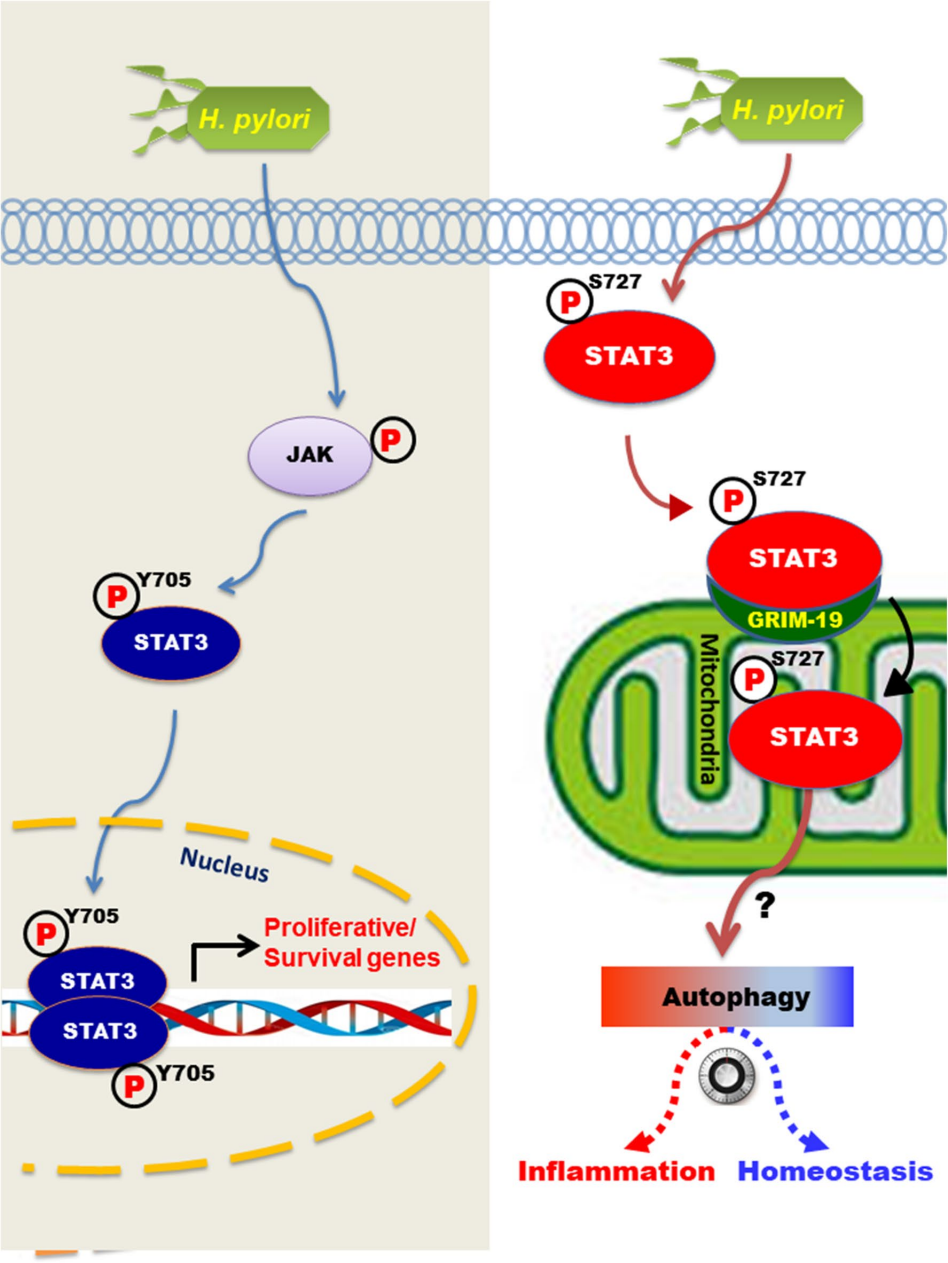
A proposed scheme illustrating the potential role of STAT3 phosphorylation on Ser727 in regulating mitochondrial autophagy. While P-STAT3^Tyr705^ localized in the nucleus of *H. pylori* infected AGS cells transactivates the target genes, STAT3 phosphorylated on Ser727 is imported into mitochondria by interacting with GRIM-19. P-STAT3^S727^ localized in mitochondria may direct autophagosome formation through an unknown mechanism. Autophagic clearance of impaired mitochondria can maintain tissue homeostasis or cause inflammation, depending on the extent of mitochondrial autophagy.

Taken together, the findings from the present study provide a novel molecular mechanism underlying host response to *H. pylori*-induced gastritis. *H. pylori* infection induces phosphorylation of STAT3 on Ser727 and subsequently its mitochondrial translocation through interaction with GRIM-19 (Fig. 6). This would facilitate the elimination of dysfunctional mitochondria formed as a consequence *H. pylori*-induced infection although it may also provoke inflammation, depending on the extent of mitophagy. Further studies will be necessary to clarify the (patho)physiologic significance of P-STAT3^Ser727^-mediated mitochondrial autophagy in *H. pylori*-induced gastritis and gastric carcinogenesis.

## Materials and methods

### Materials

RPMI-1640 medium, fetal bovine serum (FBS), penicillin , streptomycin were products of GIBCO BRL (Grand Island, NY, USA). Sheep blood agar, Gaspak™ and anaerobic jars were provided by BD Biosciences (Sparks, MD, USA). Primary antibody against actin was purchased from Sigma-Aldrich (St. Louis, MO, USA). Primary antibodies for lamin B and GRIM-19 were obtained from Santa Cruz Biotechnology (Santa Cruz, CA, USA). Antibodies against P-STAT3^Tyr705^, P-STAT3^Ser727^, total STAT3, HA, VDAC and LC3B were bought from Cell Signaling Technology (Beverly, MA, USA). Primary antibody for α-tubulin was a product from Biogenex (Fremont, CA, USA). Horseradish peroxidase (HRP)-conjugated secondary antibody was obtained from Pierce Biotechnology (Rockford, IL, USA). CCCP and DL-dithiothreitol (DTT) were purchased from Sigma-Aldrich (St. Louis, MO, USA). Alexa 588 conjugated-IgG, TRIzol®, MitoTracker, DAPI, and Lipofectamine® RNAiMAX were provided by Invitrogen (Carlsbad, CA, USA). Polyvinylidene difluoride (PVDF) membranes were supplied from Gelman laboratory (Ann Arbor, MI, USA). Protease inhibitor cocktail tablets were provided from Boehringer Mannheimm (Mannheimm, Germany). The ECL chemiluminescent detection kit was purchased from LPS solution (Daejon, South Korea). A protein assay dye (Bradford) reagent was supplied by Bio-Rad Laboratories (Hercules, CA, USA). The bicinchonic acid (BCA) protein assay reagent was obtained from Pierce Biotechnology (Rockford, IL, USA). All other chemicals used were in the purest form available commercially.

### Cell culture

AGS cells were obtained from American Type Culture Collection (Manassas, VA, USA) and cultured in RPMI-1640 medium supplemented with 10% v/v FBS and 100 units/mL penicillin and 100 µg/mL streptomycin at 37 °C in an incubator with humidified atmosphere of 95% O_2_/5% CO_2._.

### Bacteria strain and infection condition

*H. pylori* (ATCC^®^ 43504^TM^) with the typical S shape, gram-negative rods, possessing the CagA and VacA were provided in a frozen state by ATCC. A mouse adaptive strain of *H. pylori*, Sydney strain 1 (SS1)^40^ was provided by Prof. Ki-Baik Hahm of CHA University. Both strains were grown on tryptic soy agar with 5% sheep blood agar (BD Diagnostics) and Dent antibiotics supplement (Oxoid) at 37 °C under microaerophilic conditions (Campy-Pak System; BBL). AGS cells were incubated overnight in fresh serum- and antibiotic-free RPMI medium and were infected with *H. pylori* at multiplicities of infection (MOI) of 100:1.

### Western blot analysis

The cell lysates were prepared, and the protein concentration was measured as described previously^7^. The equivalent amounts of proteins (10–30 µg) were subjected to electrophoresis on 8% or 12% SDS–polyacrylamide gel and transferred to PVDF membrane. The transferred proteins were blocked in 5% fat-free dry milk in phosphate-buffered saline (PBS) containing 0.1% tween 20 (PBST) for 1 h at room temperature. Then, the membranes were incubated with primary antibodies in 3% fat-free dry milk in PBS overnight in 4 °C. Membranes were washed followed by incubation with 1:3000 dilution of respective HRP conjugated secondary antibodies for 1.5 h and again washed with PBST. Protein expressed was visualized with an ECL chemiluminescence detection kit.

### Plasmid constructs and transient transfections

The full-length human *STAT3* was amplified by RT-PCR from the total RNA obtained from AGS cells with primers 5′-ACG CTC GAG TTA TGG CCC AAT GGA ATC AG-3′ (forward) and 5′- ATT CTT ATG CGG CCG CTC ACA TGG GGG AGG TAG-3′(reverse) and subcloned into HA-tagged pCMV expression vector (addgene Plasmid #28023) as *Xho*I/*Not*I fragment. pCMV-HA-STAT3 was used as a template. STAT3 mutant constructs were generated using a site-directed mutagenesis kit (iNtRon; Seongnam, South Korea). Primer sequences used for PCR are as follows. Y705F-FP 5′- GTA GCG CTG CCC CAT TCC TGA AGA CCA AGT TT-3′ (forward) and Y705F-RP 5′-AAA CTT GGT CTT CAG GAA TGG GGC AGC GCT AC-3′(reverse). S727A-FP 5′-GAC CTG CCG ATG GCC CCC CGC ACT TTA GAT-3′ (forward) and S727A-RP 5′-ATC TAA AGT GCG GGG GGC CAT CGG CAG GTC-3′(reverse). Mutant constructs were confirmed by DNA sequence analysis. The plasmids were transfected into AGS cells using the FuGENE HD Transfection (Promega; UK), according to the optimized protocol for the AGS cells.

### Preparation of cytosolic and nuclear extracts

After *H. pylori* infection, cells were washed twice with ice-cold 1× PBS and scraped in 1 mL 1× PBS, followed by centrifugation at 1700×*g* for 5 min at 4 °C. Pellets were resuspended in hypotonic buffer A [10 mM N*-*2-hydroxyethylpiperazine*-N’-*2-ethanesulfonic acid (pH 7.9), 1.5 mM MgCl_2_, 10 mM KCl, 0.5 mM DTT and 0.2 mM phenylmethylsulfonylfluoride (PMSF)] for 15 min on ice. Ten % Nonidet P-40 was then added to final concentration of 0.1% for less than 5 min. The mixture was then centrifuged at 6000× *g* for 5 min at 4 °C. Supernatant was collected as the cytosolic extract and stored at − 80 °C. The pellets were washed twice with hypotonic buffer A and resuspended again in hypertonic buffer C [20 mM N*-*2-hydroxyethylpiperazine*-N’-*2-ethanesulfonic acid (pH 7.9), 20% glycerol, 420 mM NaCl, 1.5 mM MgCl_2_, 0.2 mM ethylenediaminetetraacetic acid, 0.5 mM DTT and 0.2 mM PMSF] for 1 h on ice and centrifuged at 18,000×*g* for 15 min at 4 °C. The supernatant containing nuclear proteins was collected and stored at − 80 °C. The protein concentrations of both fractions were determined by using the BCA protein assay reagent^7^.

### Preparation of cytosolic and mitochondrial extracts

Cytosolic extract and mitochondrial pellets were prepared by a commercial Mitochondria Isolation Kit (Thermo Fisher Scientific; Rockford, IL, USA). The mitochondrial pellets were lysed by RIPA buffer [150 mM NaCl, 1% NP-40, 0.5% sodium deoxycholate, 0.1% Tris–HCl (pH 8.0) and one tablet of protease inhibitor cocktail]. The protein concentrations of cytosolic and mitochondrial extracts were determined using the BCA protein assay reagent.

### Immunocytochemical analysis

AGS cells were infected with *H. pylori* for 12 h. After the cells were incubated with MitoTracker probe (1 nM) in prewarmed staining solution for 30 min, samples were fixed with cold 95% MeOH/5% acetic acid for 10 min at 4 °C. Then samples were permeabilized with 0.2% Triton X-100 for 5 min at room temperature and blocked with 5% bovine serum albumin in PBST for 1 h at room temperature. Samples were incubated with primary antibody specific for phospho-STAT3^Ser727^ or LC3B overnight at 4 °C, followed by incubation with fluorescein isothiocyanate-goat anti-rabbit IgG secondary antibody for 1 h at room temperature. Nuclear-staining was performed with DAPI for 5 min at room temperature^7^. Images were assessed under a fluorescent microscopy.

### Transmission election microscopy

AGS cells with or without *H. pylori* infection were collected and fixed at room temperature for 4 h with karnovsky’s fixation. The cells were washed with 0.05 M sodium cacodylate buffer and postfixed for 2 h with 1% osmium tetroxide in 0.1 M cacodylate buffer. After fixation, the cells were stained with 0.5% uranyl acetate for overnight. The stained cells were dehydrated in graded alcohol and embedded in spurr’s resine. Then, ultra-thin sections (100 nm) were prepared, stained with uranyl acetate and lead citrate, and examined under a transmission electron microscope (JEM1010, JEOL, Japan).

### Animal treatment

The protocol for animal treatments is the same as reported previously^7^. All animal experiments were carried out in accordance with the 8th edition of the Guide for the Care and Use of Laboratory Animals (National Research Council, 2011), and the protocol was approved by the Institutional Animal Care and Use Committee (IACUC) of Seoul National University (SNUIBC-R141017-1).

### Preparation of whole lysates from mouse stomach tissue

Part of excised stomach tissues were homogenized in ice-cold lysis buffer [150 mM NaCl, 0.5% Triton-X 100, 50 mM Tris–HCl (pH 7.4), 20 mM ethyleneglycoltetra-acetic acid, 1 mM DTT, 1 mM Na_3_VO_4_ and protease inhibitors, 1 mM PMSF and ethylenediaminetetra-acetic acid-freecocktail tablet] followed by periodical vortex mixing for 30 min at 0 °C. Lysates were centrifuged at 14,000 rpm for 15 min at 4 °C. Supernatants were collected and stored at − 80 °C until use.

### Histopathological examinations

After 4 weeks of *H. pylori* infection, mouse stomachs were subjected to histological analysis. The tissues were fixed with 10% formalin before being embedded in paraffin. Each tissue blot Sections (4 µm) was stained with hematoxylin and eosin (H&E).

### Immunofluorescence analysis

The dissected stomach tissues were prepared as previously^7^ for measuring the expression patterns of P-STAT3^Ser727^, LC3 and VDAC. Four-μm sections of 10% formalin-fixed, paraffin-embedded tissues were placed on glass slides and deparaffinized twice with xylene and rehydrated through graded alcohol bath. The deparaffinized sections were heated by using microwave and boiled twice for 10 min in 10 mM citrate buffer (pH 6.0) for antigen retrieval. Then the samples were incubated with 0.2% Triton for 45 min for permeabilization. To diminish nonspecific staining, each section was blocked for 1 h with 3% bovine serum albumin in PBS. For the detection of respective protein expression, slides were incubated with P-STAT3^Ser727^, LC3 and VDAC antibodies at 4 °C for overnight and Alexa Fluor (Invitrogen) anti-mouse or rabbit (1:1,000) secondary antibody at room temperature for an additional 1 h. The slides were mounted by prolong antifade with DAPI (Invitorgen).

### Statistical analysis

Data from three independent experiments at least were expressed as the mean ± S.D. The statistical significance of differences between two groups was evaluated using Student’s *t* test. Analysis was performed using Sigmaplot (Version 10). Statistical significance was accepted at *p* < 0.05, unless otherwise indicated.

## Supplementary information

Supplementary information.

## Data Availability

The datasets generated during and/or analyzed during current study are available from the corresponding author on reasonable request.
