# Supplementary material for: Helicobacter pylori infection induces STAT3 phosphorylation on Ser727 and autophagy in human gastric epithelial cells and mouse stomach
Source: Sci Rep. 2020 Sep 24;10:15711. doi: 10.1038/s41598-020-72594-3 (PMC7519032; doi:10.1038/s41598-020-72594-3)
Supplement: Supplementary file 1 — Supplementary information. [file 41598_2020_72594_MOESM1_ESM.pdf]

Methods to Supplementary Figure

siRNA transient transfection

AGS cells were transfected with Grim19-siRNA or LC3B-siRNA or scrambled siRNA using the transfection reagent Lipofectamine® RNAiMAX for 24 h according to manufacturer's instructions. The sequences of human Negative-siRNA, Grim19-siRNA and LC3B-siRNA were 5'-CCUCGUGCCGUUCCAUCAGGUAGUU-3', 5'-GGCACUGGAGCAUAAUGAAUU-3' and 5'-GGCACUGGAGCAUAAUGAAUU-3', all of them were purchased from Genolution Pharmaceuticals, Inc. (Seoul, South Korea). Transfected cells were then treated with *H. pylori* for 12 h and harvested for the next experiments.

Immunoprecipitation of GRIM19 and P-STAT3^{Ser727} in mitochondrial extracts

The mitochondrial pellets were isolated by a commercial Mitochondria Isolation Kit. The pellets were resuspended in 1x NET-NL buffer [0.05 M Tris-HCl (pH 7.5), 5 mM EDTA, 0.15 M NaCl, 1 mM DTT, 0.5% NP-40, 0.2 mM PMSF and one tablet of protease inhibitor cocktail] for 30 min on ice. Then the lysates were centrifuged at 10,000 x *g* for 15 min at 4°C. Supernantant was collected and measured the protein concentration. The cellular proteins (5-10 μg) were subjected to immunoprecipitation by P-STAT3^{Ser727} primary antibody at 4°C for overnight followed by the addition of protein A/G-agarose beads suspension (40 μl) and additional shaking for 4 h at the same condition. After centrifuge at 10,000 x *g* for 1 min, immuoprecipitated beads were collected by discarding the supernatant and washed with 1x NET-NL buffer. After final washing, immunoprecipitate was resuspendend in 40 μl of 2x SDS electrophoresis sample buffer and boiled for 10 min. The supernantant (30 μl) from each sample were collected after centrifugation and loaded on SDS-PAGE gels. The western blots were developed with antibody against Grim19.

Legend to Supplementary Figures

Figure S1. Role of Ser727 phosphorylated STAT3 in LC3B expression in AGS cells. STAT3 silenced AGS cells were transfected with mock, wilde type (WT) or Ser727 mutant (SA) vector for 24 h. Cells were then treated with CCCP (1 μM) for 1 h, and expression of LC3B was measured by Western blot analysis as described in Materials and Methods.

Figure S2. *H. pylori*-induced mitochondrial translocation of P-STAT3^{Ser727} is mediated through interaction with Grim19. (A) AGS cells were infected by *H. pylori* and harvested at indicated time points, followed by mitochondria isolation. Mitochondrial lysates were immunoprecipitated with a P-STAT3^{Ser727} antibody, followed by immunoblot analysis to measure Grim19 bound to P-STAT3^{Ser727}. (B) AGS cells were transfected with WT and serine dominant mutant vector and then inoculated with *H. pylori* for 12 h. Samples were immunoprecipitated with an HA antibody, followed by Western blot analysis to measure Grim19. (C) AGS cells were transfected with 20 nM of siRNA targeted against Grim19 prior to *H. pylori* infection. Then, the cells were harvested at 12 h later and subjected to lysis followed by Western blot analysis to determine P-STAT3^{Ser727} in mitochondrial and cytosolic extracts. VDAC and α-tubulin are mitochondrial and cytoplasmic protein markers, respectively. (D) Grim19 expression in *H. pylori*-infected AGS cells was silenced by a specific siRNA as described above. The cell lysates were subjected to Western blot analysis to detect P-STAT3^{Ser727}, LC3B and Grim19. (E) AGS cells transfected with si-Grim19 (20 nM) prior to *H. pylori* infection. Then, the cells were harvested and subjected to lysis followed by Western blot analysis to determine COX-2 and Grim19.

Figure S3. The potential link between mitophagy and inflammation. (A) Western blot analysis of COX-2 and Actin in *H. pylori*-infected AGS cells. (B) Western blot analysis of COX-2 and actin in CCCP-treated AGS. (C) AGS cells were transfected with si-LC3 (20 nM) prior to *H. pylori* infection. Then the cells were harvested and subjected to lysis followed by Western blot analysis to determine expressions of COX-2 and LC3. (D) AGS cells were treated with an autophagy inhibitor, 3 MA for 30 min prior to *H. pylori* infection. The cells were then harvested and subjected to lysis followed by Western blot analysis to determine expression of COX-2 and actin. (E) Western blot analysis of COX-2 in WT and Serine dominant negative vector transfected AGS cells with or without *H. pylori* infection.

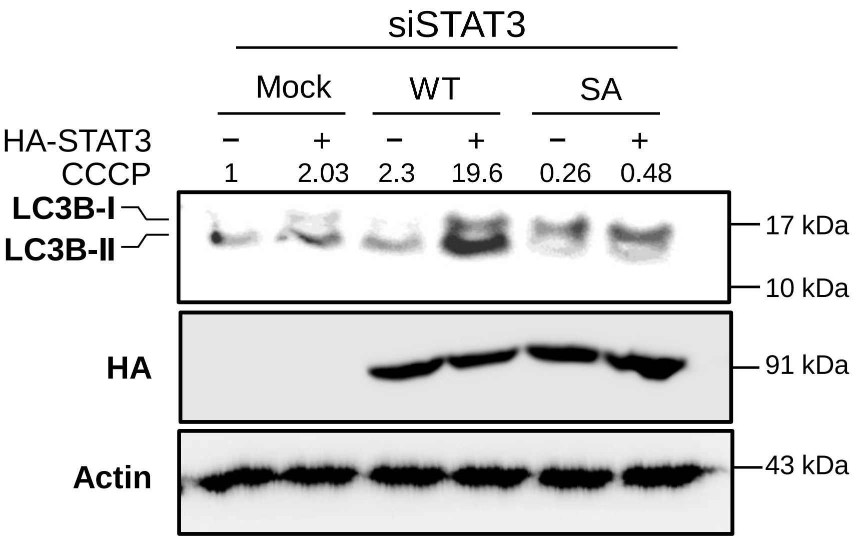

Supplementary Fig. S1

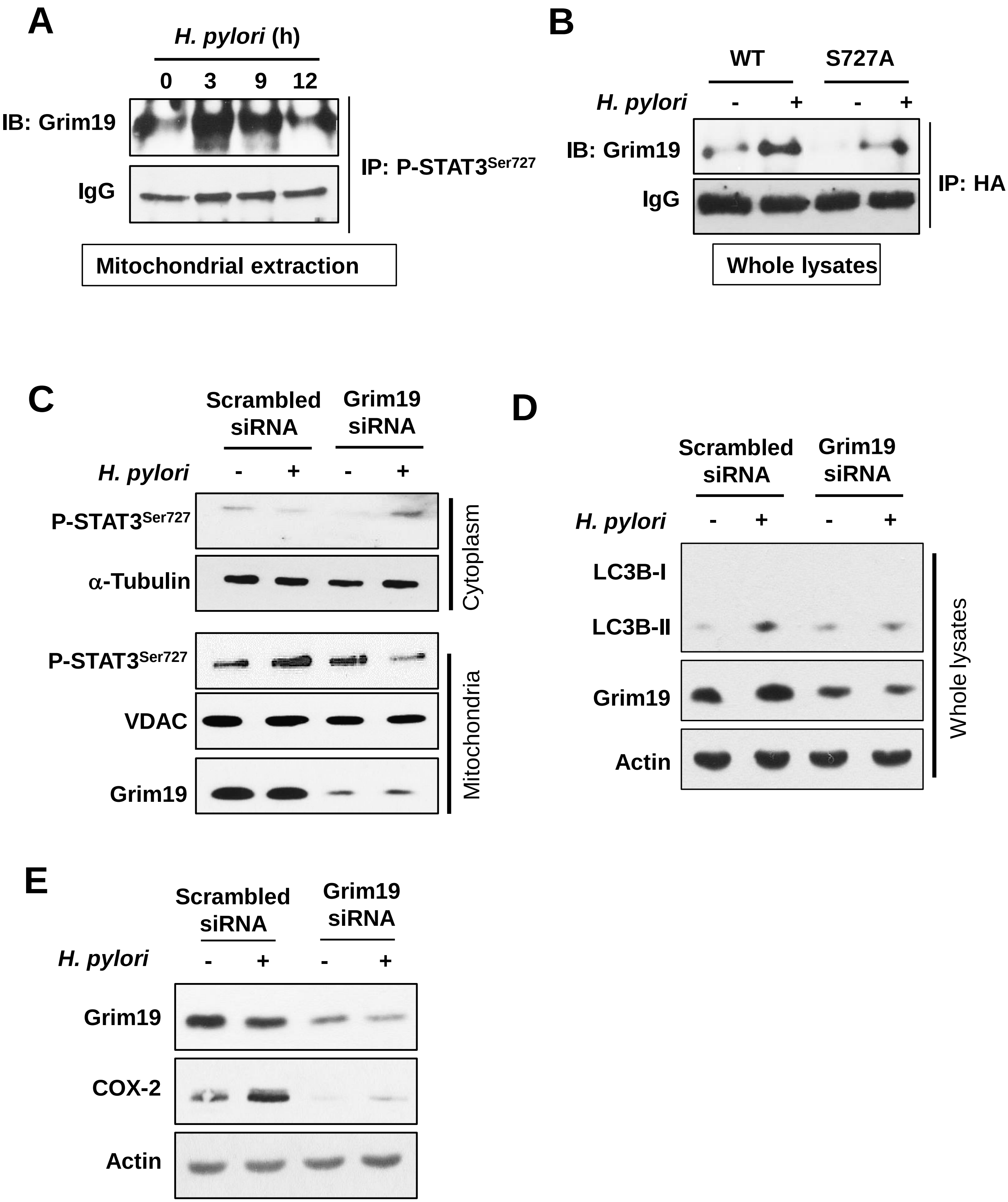

Supplementary Fig. S2

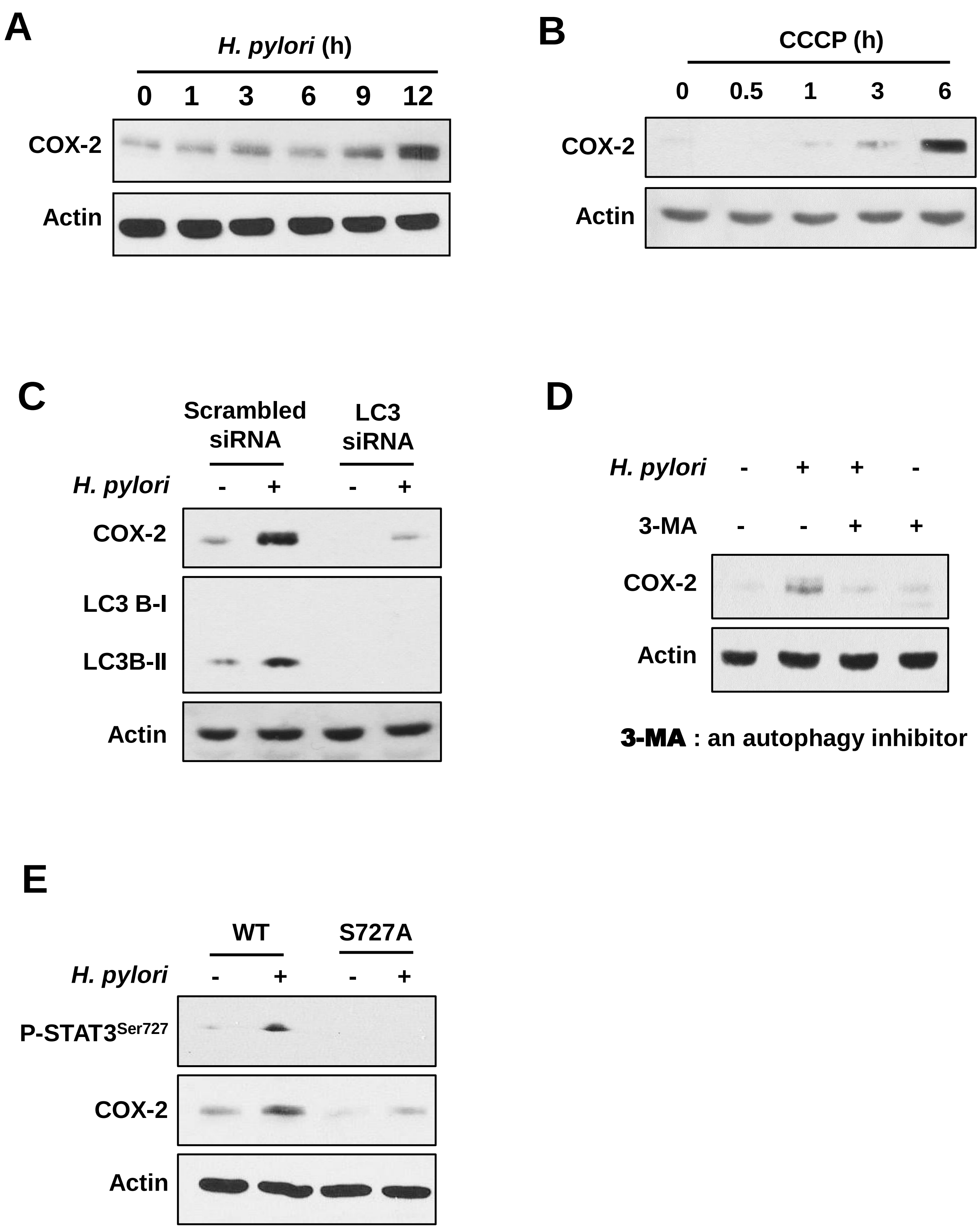

Supplementary Fig. S3

a

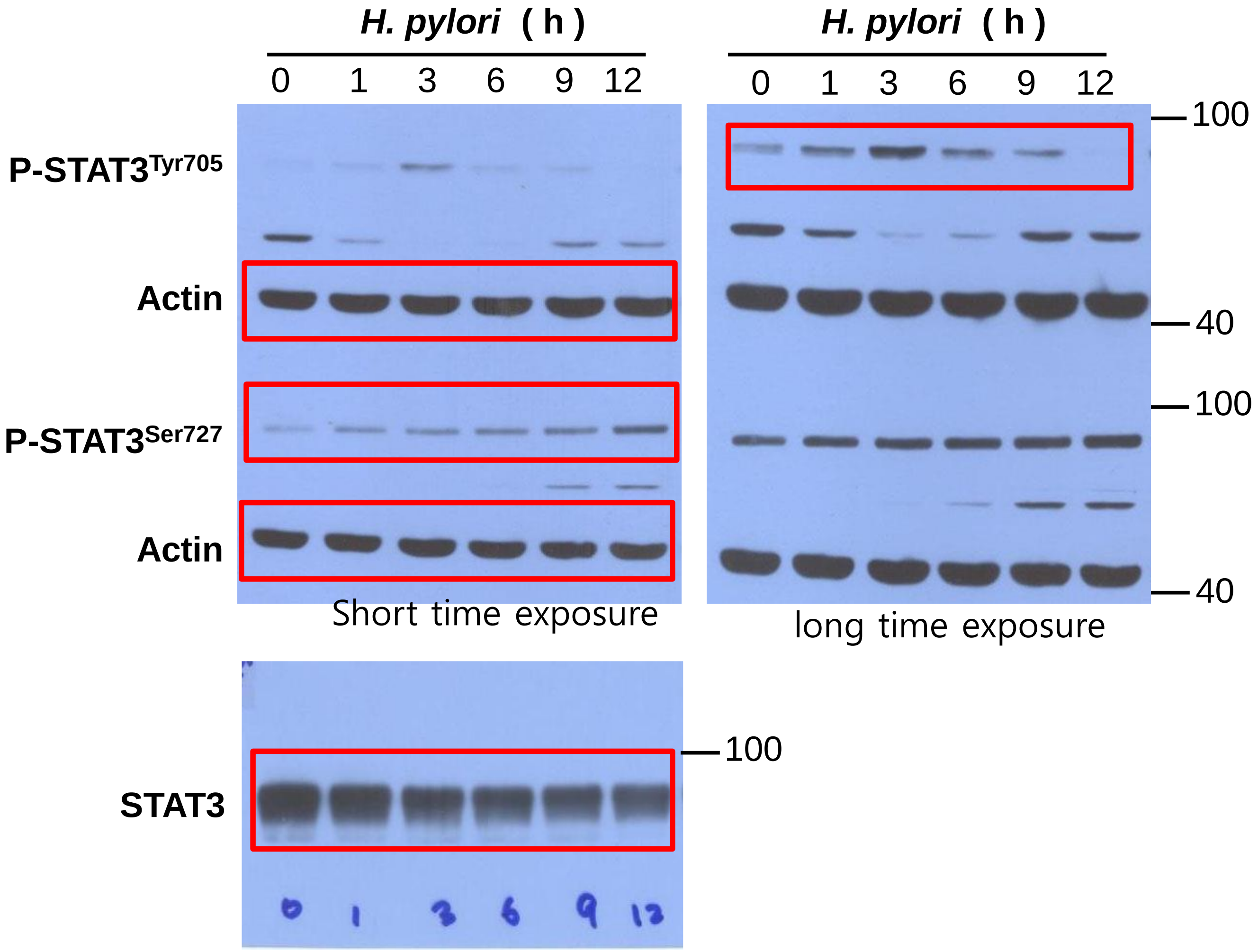

Supplementary Fig. S4. Whole blot data for Fig. 1. Indicated parts surrounded by red lines are shown in corresponding figures.

b

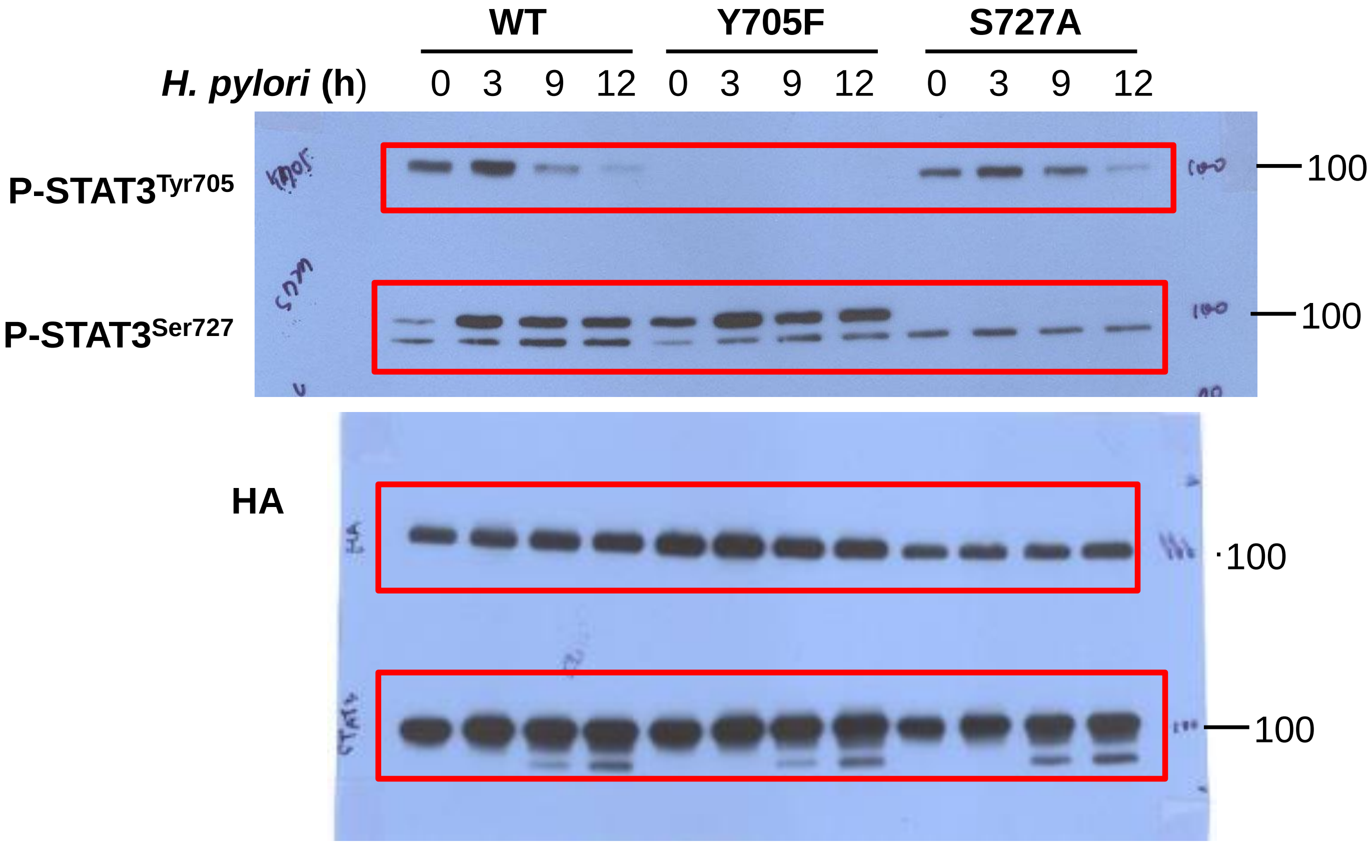

Supplementary Fig. S4 (continued). Whole blot data for Fig. 1. Indicated parts surrounded by red lines are shown in corresponding figures.

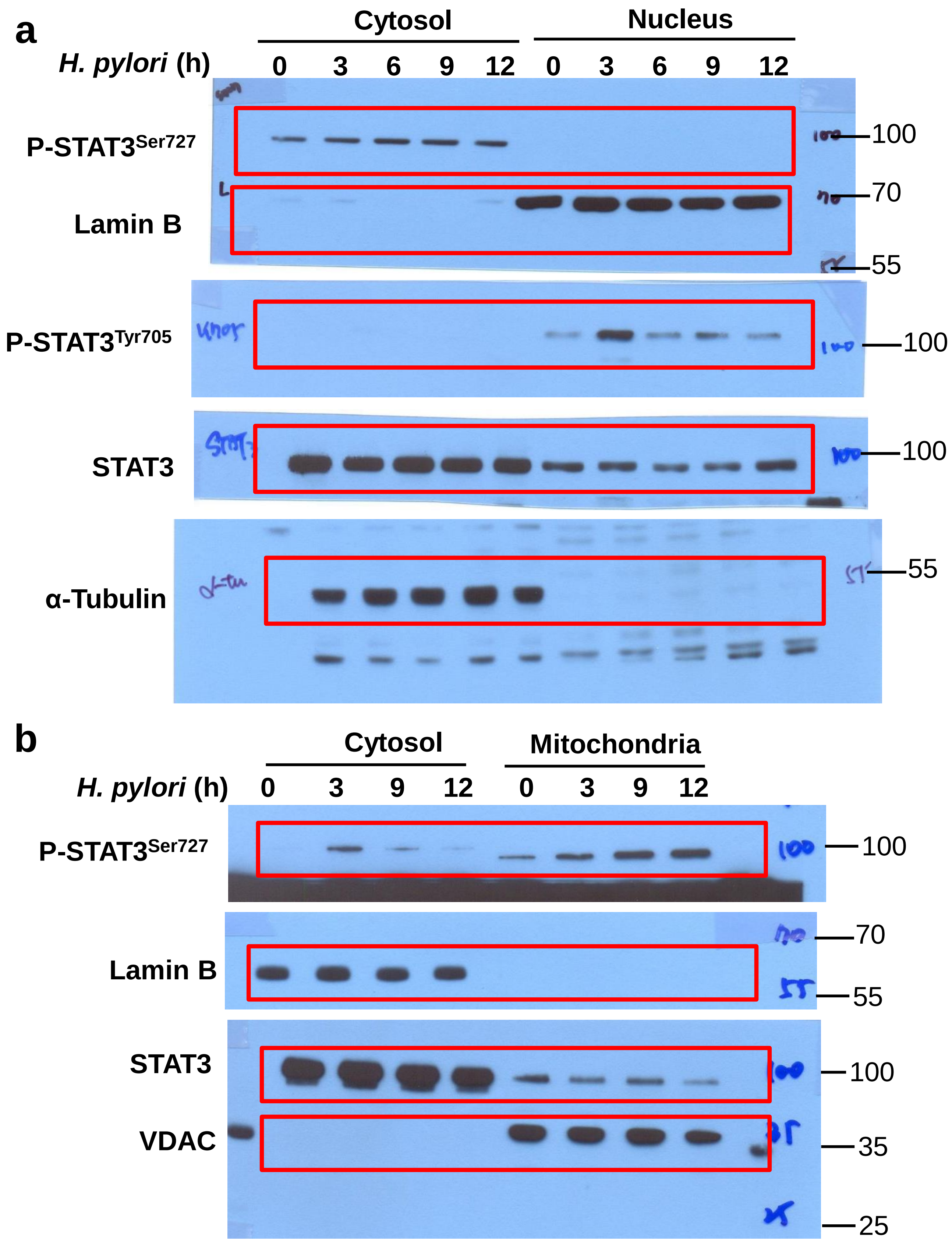

Supplementary Fig. S5. Whole blot data for Fig. 2. Indicated parts surrounded by red lines are shown in corresponding figures.

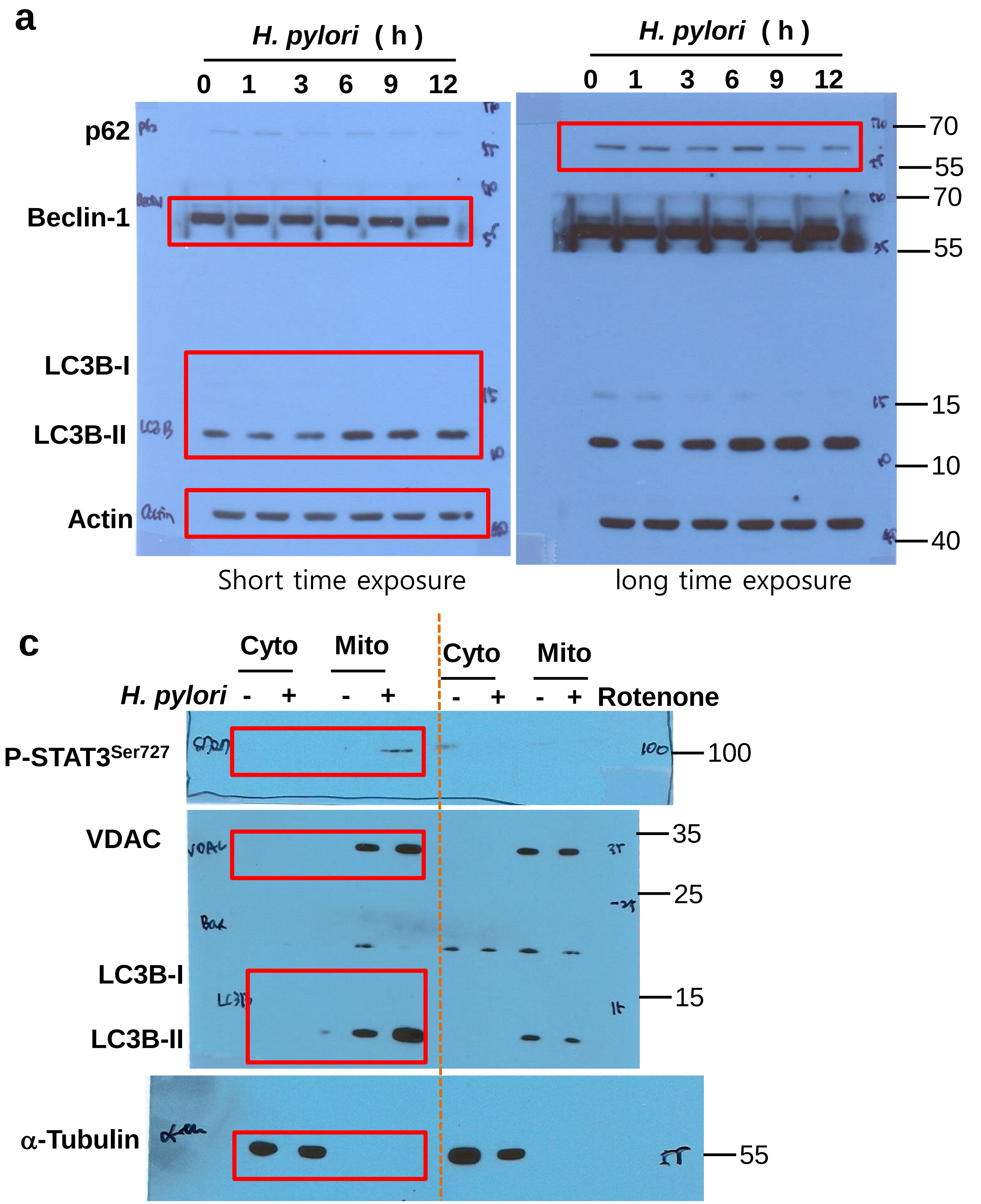

Supplementary Fig. S6. Whole blot data for Fig. 3. Indicated parts surrounded by red lines are shown in corresponding figures.

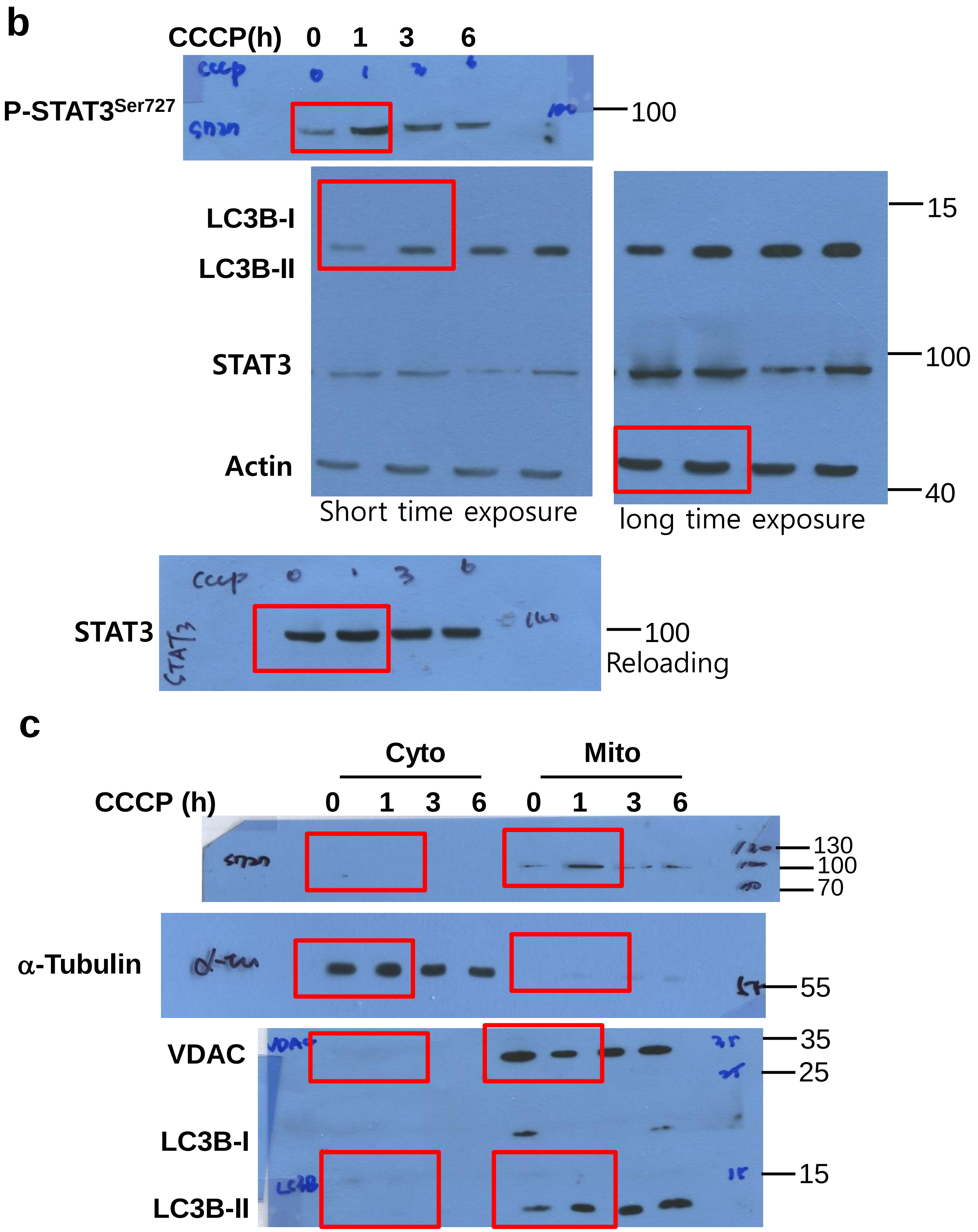

Supplementary Fig. S7. Whole blot data for Fig. 4. Indicated parts surrounded by red lines are shown in corresponding figures.

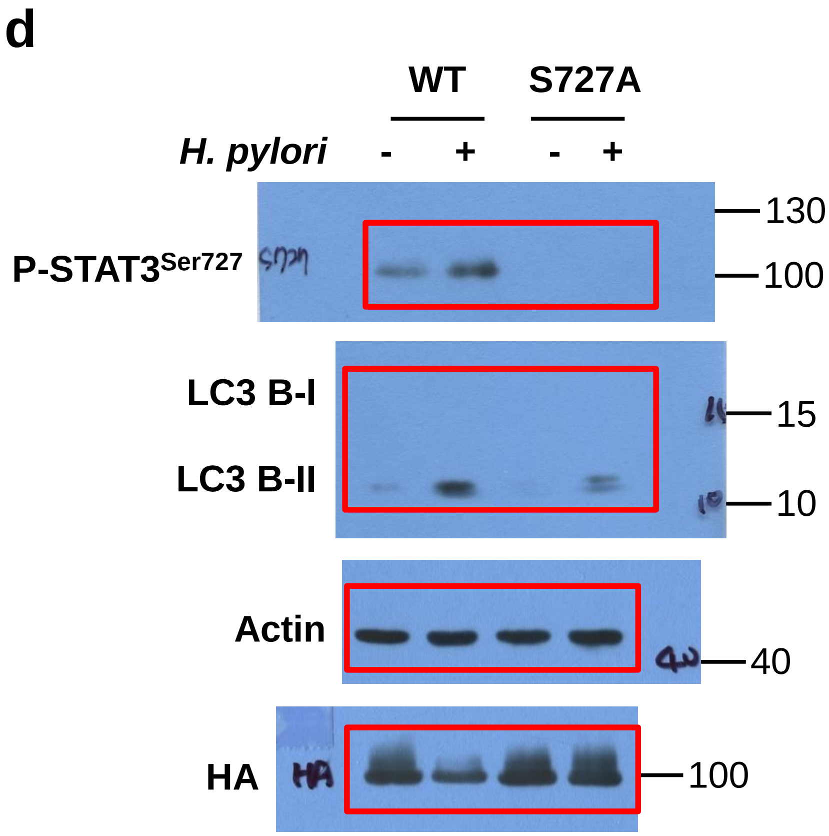

Supplementary Fig. S7 (continued). Whole blot data for Fig. 4. Indicated parts surrounded by red lines are shown in corresponding figures.

a

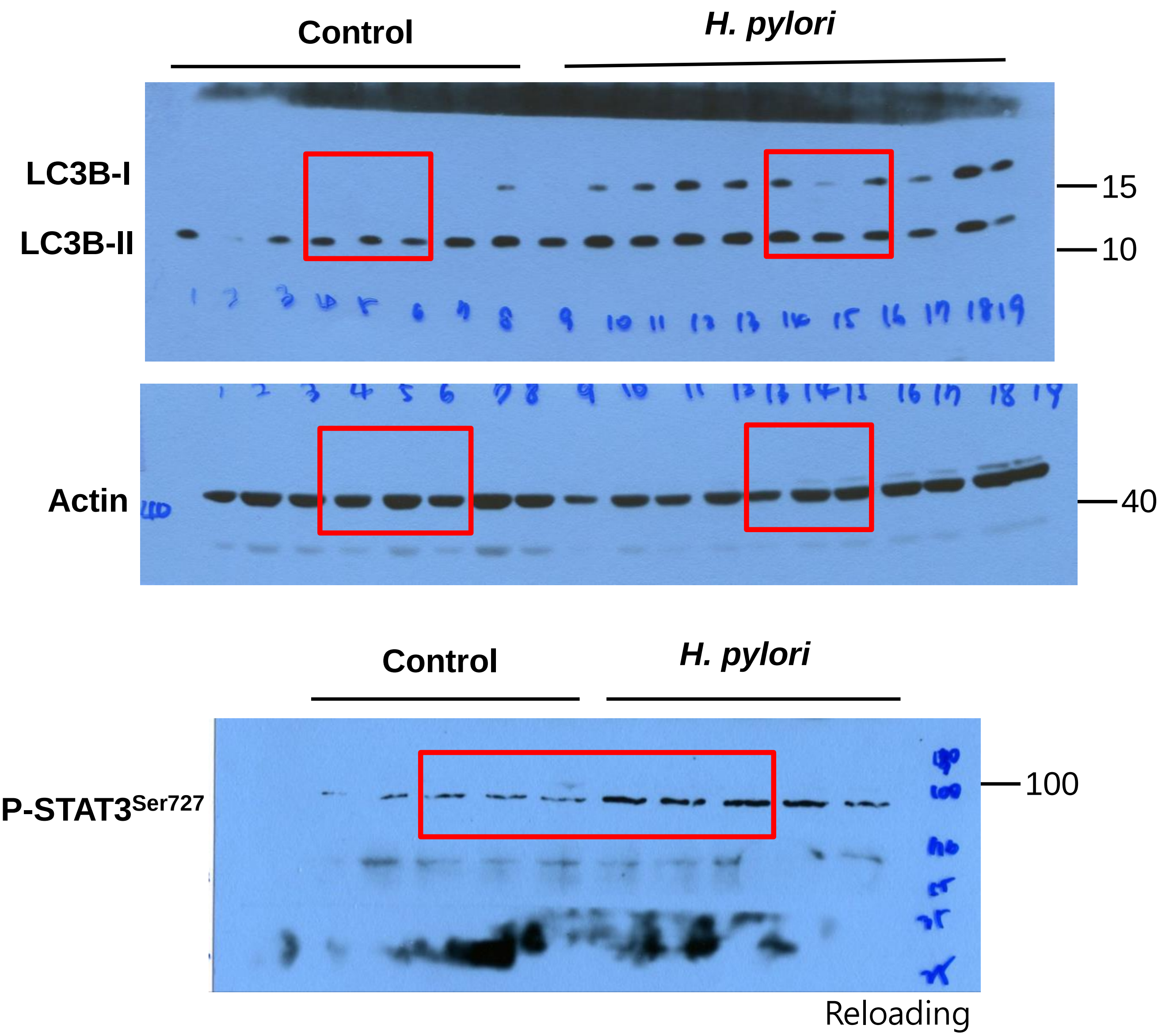

Supplementary Fig. S8. Whole blot data for Fig. 5. Indicated parts surrounded by red lines are shown in corresponding figures.

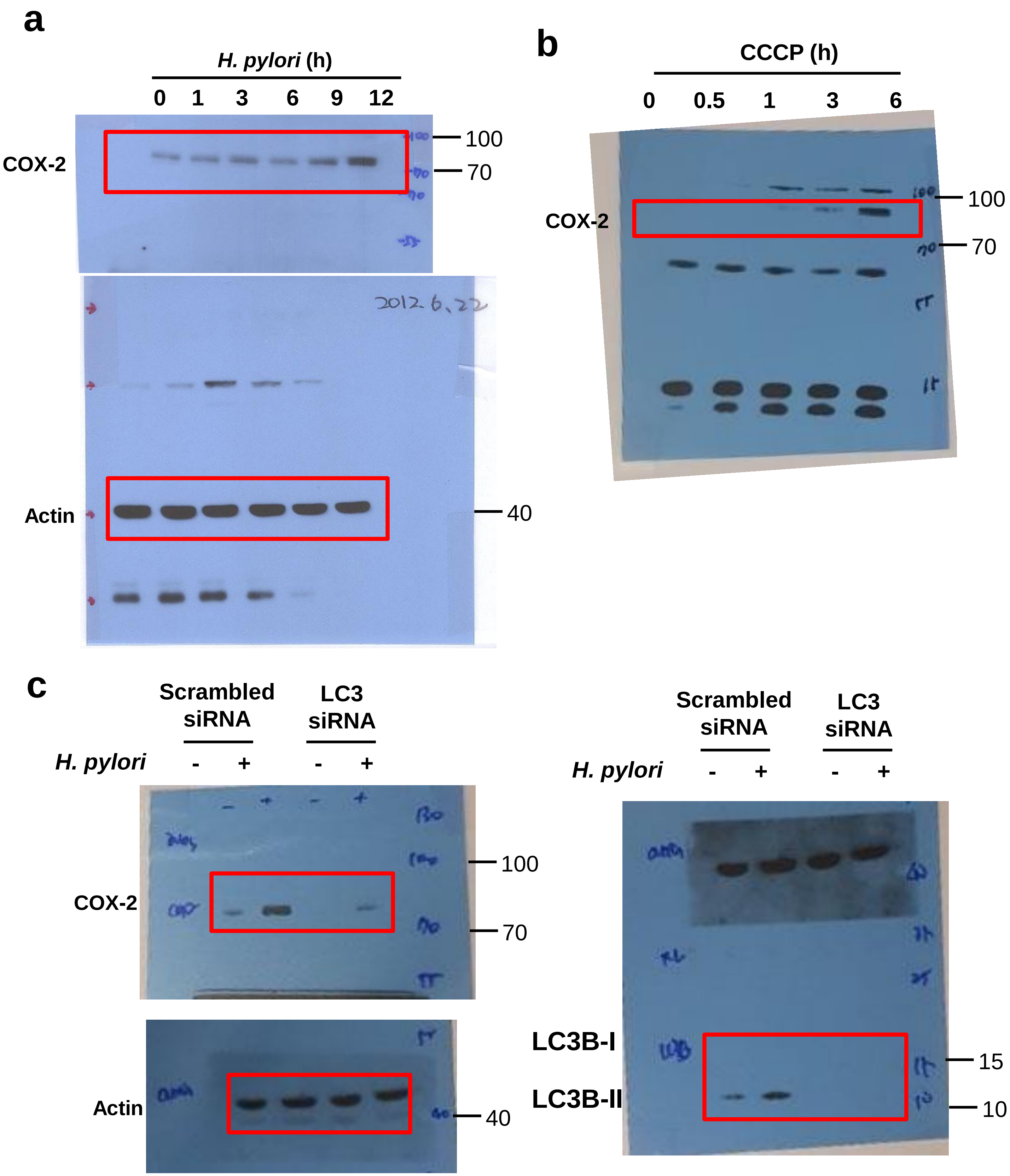

Supplementary Fig. S9. Whole blot data for Supplementary Fig. 2.
Indicated parts surrounded by red lines are shown in corresponding figures

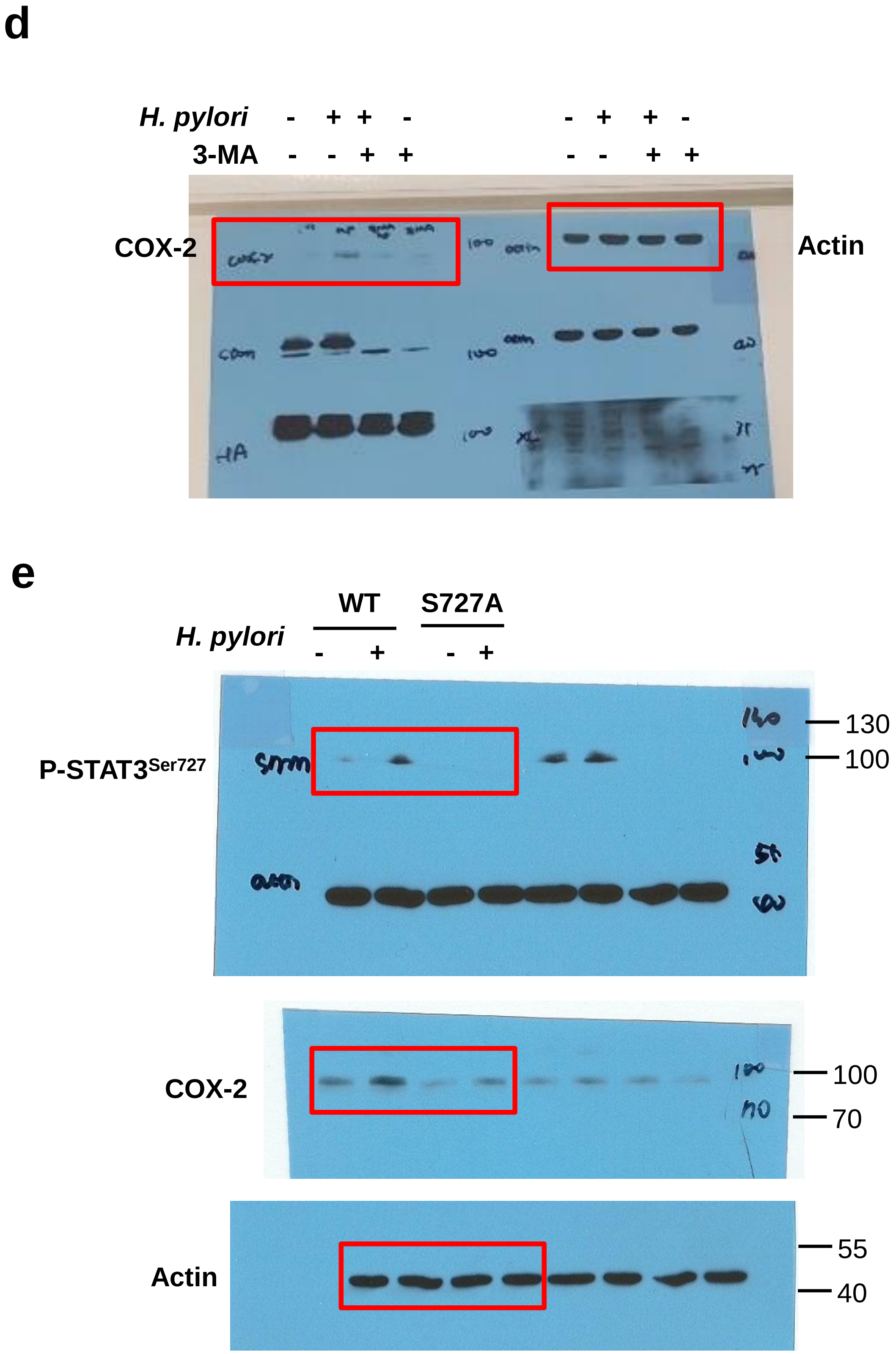

Supplementary Fig. S9 (continued). Whole blot data for Supplementary Fig. 2. Indicated parts surrounded by red lines are shown in corresponding figures.

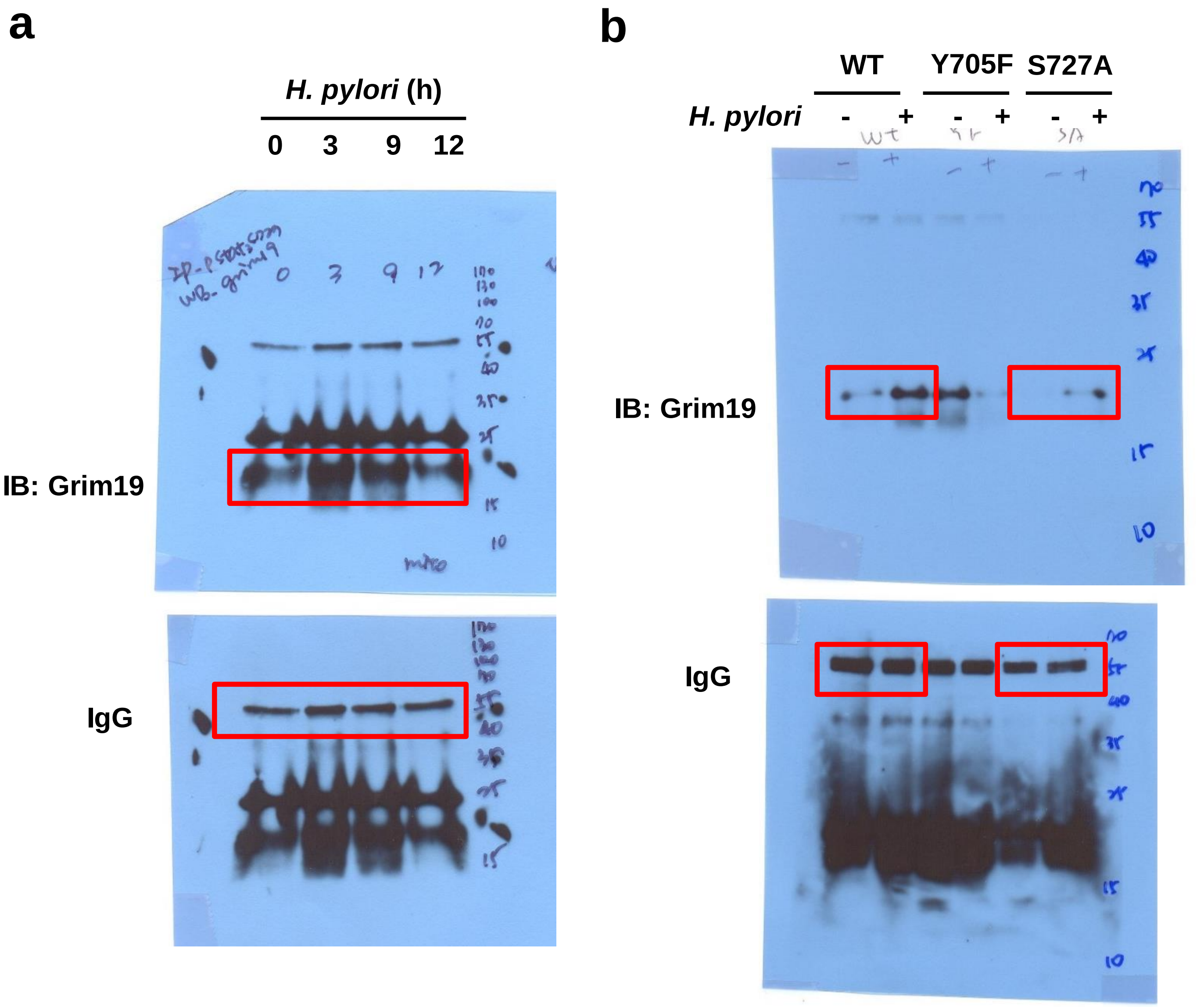

Supplementary Fig. S10. Whole blot data for Supplementary Fig. 3. Indicated parts surrounded by red lines are shown in corresponding figures

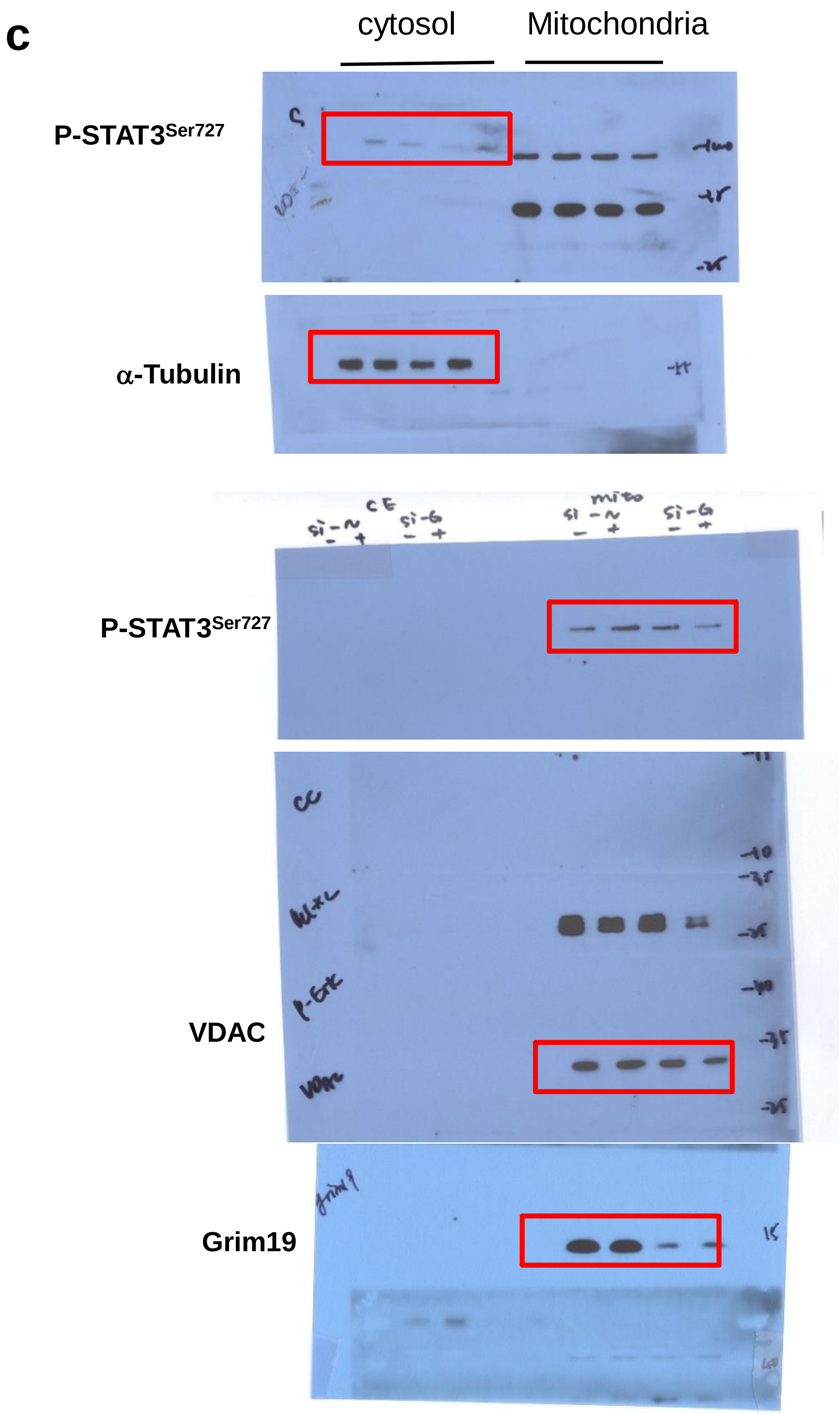

Supplementary Fig. S10 (continued). Whole blot data for Supplementary Fig. 3. Indicated parts surrounded by red lines are shown in corresponding figures.

d

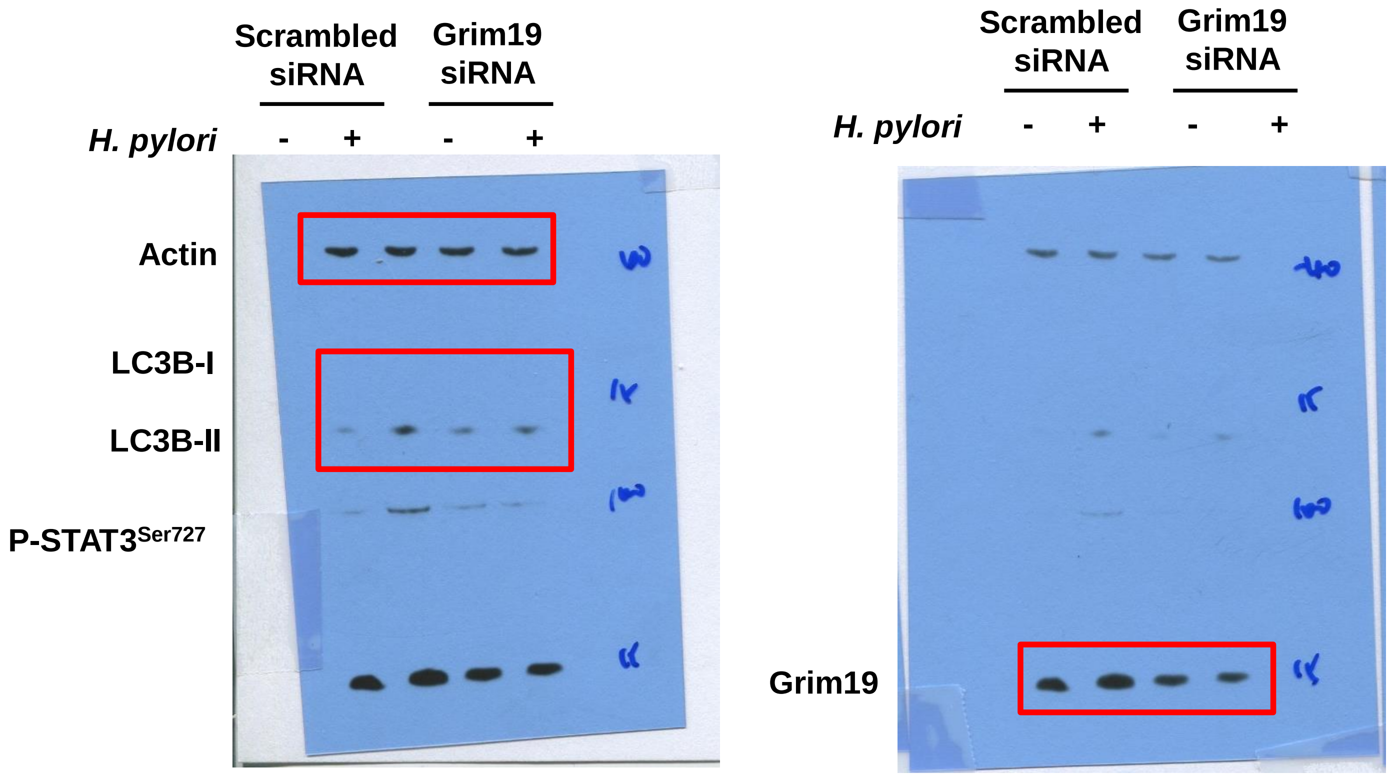

e

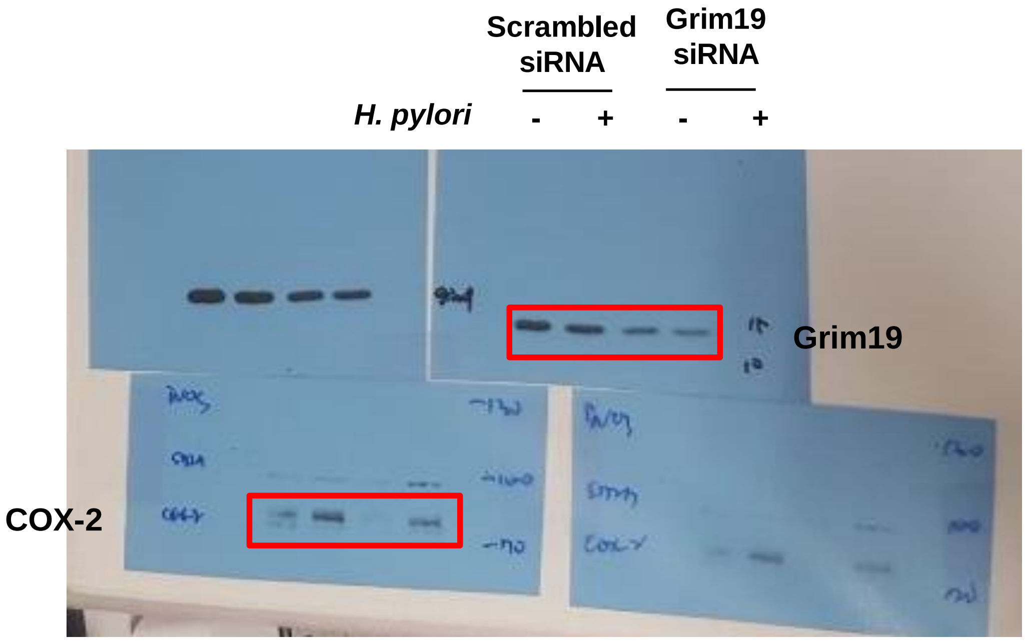

Supplementary Fig. S10. Whole blot data for supplementary figure 3. Indicated parts surrounded by red lines are shown in corresponding figures (continued)
